# CD8^+^ tissue-resident memory T cells promote liver fibrosis resolution by inducing apoptosis of hepatic stellate cells

**DOI:** 10.1038/s41467-021-24734-0

**Published:** 2021-07-22

**Authors:** Yuzo Koda, Toshiaki Teratani, Po-Sung Chu, Yuya Hagihara, Yohei Mikami, Yosuke Harada, Hanako Tsujikawa, Kentaro Miyamoto, Takahiro Suzuki, Nobuhito Taniki, Tomohisa Sujino, Michiie Sakamoto, Takanori Kanai, Nobuhiro Nakamoto

**Affiliations:** 1 Division of Gastroenterology and Hepatology, Department of Internal Medicine, Keio University School of Medicine, Tokyo, Japan; 2 Mitsubishi Tanabe Pharma Corporation, Kanagawa, Japan; 3 Department of Pathology, Keio University School of Medicine, Tokyo, Japan; 4 Japan Agency for Medical Research and Development, AMED, Tokyo, Japan

**Keywords:** Chemokines, Cytotoxic T cells, Non-alcoholic steatohepatitis

## Abstract

Non-alcoholic steatohepatitis (NASH) is a leading cause of chronic liver disease that can progress to liver fibrosis. Recent clinical advance suggests a reversibility of liver fibrosis, but the cellular and molecular mechanisms underlying NASH resolution remain unclarified. Here, using a murine diet-induced NASH and the subsequent resolution model, we demonstrate direct roles of CD8^+^ tissue-resident memory CD8^+^ T (CD8^+^ Trm) cells in resolving liver fibrosis. Single-cell transcriptome analysis and FACS analysis revealed CD69^+^CD103^**−**^CD8^+^ Trm cell enrichment in NASH resolution livers. The reduction of liver CD8^+^ Trm cells, maintained by tissue IL-15, significantly delayed fibrosis resolution, while adoptive transfer of these cells protected mice from fibrosis progression. During resolution, CD8^+^ Trm cells attracted hepatic stellate cells (HSCs) in a CCR5-dependent manner, and predisposed activated HSCs to FasL-Fas-mediated apoptosis. Histological assessment of patients with NASH revealed CD69^+^CD8^+^ Trm abundance in fibrotic areas, further supporting their roles in humans. These results highlight the undefined role of liver CD8^+^ Trm in fibrosis resolution.

## Abstract (web summary)

The cellular and molecular mechanisms underlying the resolution of non-alcoholic steatohepatitis remain incompletely understood. Here the authors report a single cell-based analysis that identified CD8 + tissue-resident memory T cells, which contribute to resolution of liver fibrosis potentially via elimination of hepatic stellate cells through Fas-mediated cytotoxicity.

## Introduction

Non-alcoholic fatty liver disease (NAFLD) is currently the leading cause of chronic liver disease worldwide^1^. NAFLD ranges from isolated hepatic steatosis to non-alcoholic steatohepatitis (NASH) characterized by lobular inflammation, and hepatocyte ballooning^2^. Up to one third of patients with NAFLD progress to advanced fibrosis, leading to increased liver-related mortality owing to decompensated cirrhosis or development of hepatocellular carcinoma^3^. Accumulating evidence indicates that immune cells play a critical role in the development of NASH^4,5^, which is preceded by multiple bouts of liver inflammation induced by lipotoxicity^6^, gut-derived molecules^7^, and oxidative stress^8^. Repeated hepatocyte destruction creates an inflammatory milieu in which infiltrating immune cells release a myriad of cytokines and chemokines, leading to hepatic stellate cell (HSC) activation and extracellular matrix accumulation, thereby causing tissue fibrosis. Complex immune networks play a key role to maintain the homeostasis in the liver, and the balance between HSC activation and deactivation defines the overall state of liver fibrosis^9,10^. Although advanced liver fibrosis with activated HSCs has been considered to be irreversible, recent clinical evidence demonstrating substantial fibrosis resolution following successful antiviral treatment in patients with chronic hepatitis B virus (HBV) or hepatitis C virus (HCV) infection overturned this dogma^11^. Accordingly, significant efforts have been made to control fibrosis progression through inflammatory milieu modulation by targeting specific chemokines^12^, cytokines^13^, or immune cells^14^. Meanwhile, genuine fibrinolytic factors or immune cells in NASH livers have not been identified in part due to the dearth of appropriate approach to examine the cellular and molecular networks involved in the resolution process. In particular, although both pathogenic and suppressive properties of CD8^+^ T cells have been highlighted in NASH progression^5,15–18^, functional and phenotypical characteristics of intrahepatic CD8^+^ T cells associated to NASH resolution are largely unknown.

In the current study, a high-fat high-cholesterol (HFHC) diet-induced murine NASH model followed by a switch to normal diet is used to examine the dynamic change of intrahepatic immune cells during NASH development and resolution. By combining a single-cell transcriptome and parabiotic analysis, we here identify a unique subset of CD8^+^ T cells with a resident memory phenotype (CD8^+^ Trm) that promote recovery from liver fibrosis.

## Results

### NASH resolution model in mice

**Fig. 1:**
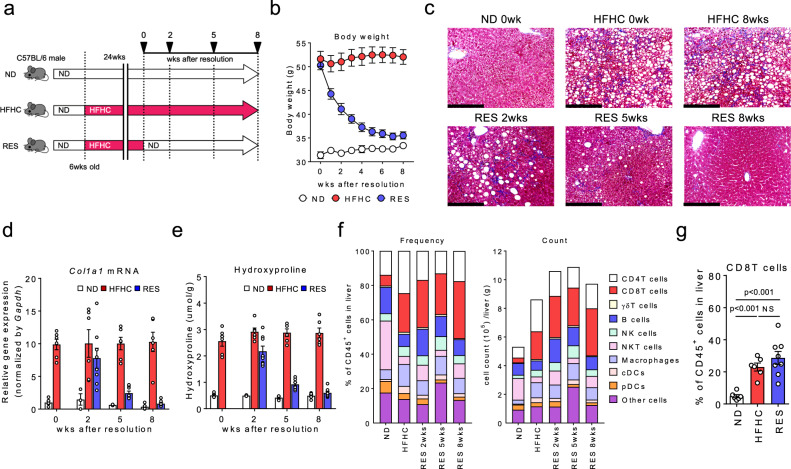
Abundance of CD8^+^ T cells in NASH resolution livers. **a** Study design: male C57BL/6 mice were fed a ND or HFHC diet for 24 weeks. After 24 weeks, HFHC diet-fed mice were either continued on a HFHC diet (HFHC group) or switched to ND (RES group) for 2, 5, or 8 weeks. **b** Body weight change following the diet switch (*n* = 7 mice for each group). **c** Representative photomicrographs of Masson trichrome staining of liver sections. Scale bars: 200 µm. **d** *Col1a1* mRNA levels in the whole liver. **e** Hydroxyproline levels in the liver (**d**, **e**: *n* = 3–6 mice for ND group, *n* = 6 mice for HFHC group, and *n* = 8 mice for RES group). **f** Frequency (left) and absolute numbers (right) of the indicated immune cells in CD45^+^ liver MNCs (*n* = 3 mice for ND, and HFHC groups, and *n* = 4 mice for RES groups). **g** Frequency of CD8^+^ T cells in CD45^+^ liver MNCs at week 8 following the diet switch (*n* = 6 mice for ND, and HFHC groups, and *n* = 8 mice for RES group). Data are presented as mean ± SEM. One-way ANOVA with Tukey’s multiple comparisons post-hoc test was applied. Source data are provided as a Source data file.

High-fat and high-cholesterol (HFHC) diet-induced NASH model recapitulates the main characteristics of human NASH in elevated liver enzymes (Supplementary Fig. 1a), body weight (Supplementary Fig. 1b), histopathology with inflammatory infiltrates of the liver, and glucose metabolism. To explore key features associated with the resolution process in liver fibrosis, we here applied a unique murine model, in which mice were fed HFHC diet for 24 weeks, and then continued with the HFHC diet (HFHC mice) or switched to a normal diet (RES mice) (Fig. 1a). We confirmed that RES mice recovered to the original condition 8 weeks after the diet switch (Fig. 1b, c and Supplementary Fig. 1c–e). Note, liver fibrosis (Fig. 1d, e) as well as lipid and glucose metabolism (Supplementary Fig. 1f, g) were almost fully recovered by the time point. Next, to explore the immunological characteristics associated with NASH fibrosis/resolution, we examined detailed immune profiles in the liver by flowcytometry (Supplementary Fig. 1h, i). We noted that both the frequency and number of intrahepatic CD8^+^ T cells remained significantly high in RES mice compared to normal diet (ND)-fed mice (ND mice), while other cell subsets tended to return to the normal condition (Fig. 1f, g and Supplementary Fig. 1i, j). Of note, the frequency of Ly-6c^low^CD11b^+^ macrophages, known as a fibrinolytic immune subset in other models, was not increased in the resolution phase (Supplementary Fig. 1i, j).

### Involvement of liver CD8^+^ Trm cells in liver fibrosis resolution

**Fig. 2:**
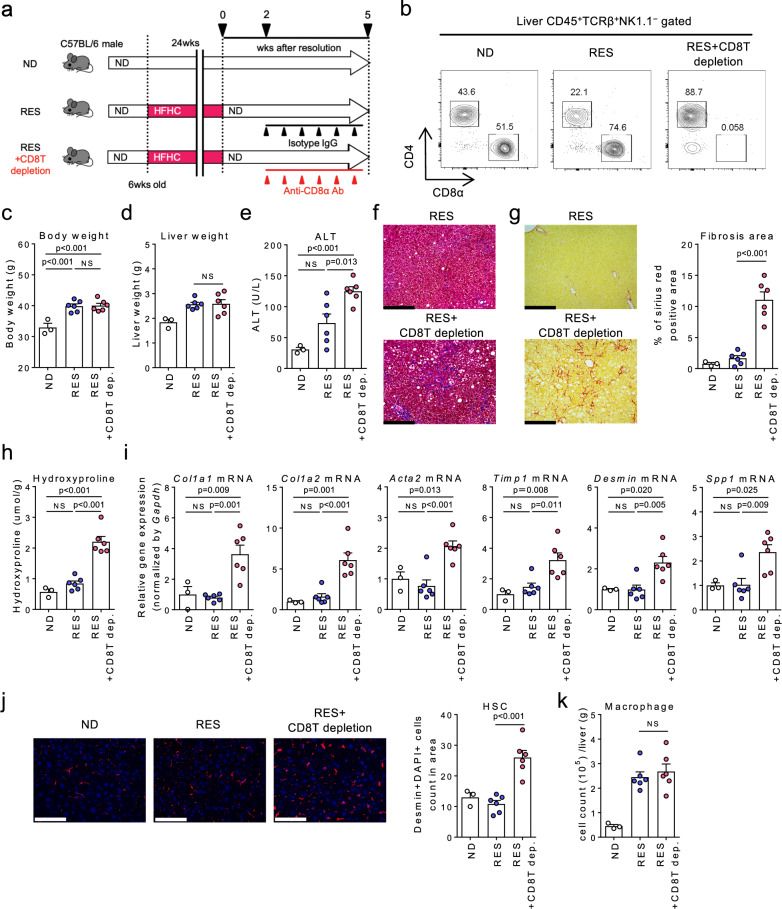
CD8^+^ T cells play a direct role in NASH resolution. **a** Study design: resolution induced mice were treated with anti-CD8α antibody (RES + CD8T depletion group) or the isotype control (RES group) once every 3 days for 3 weeks starting from week 2 after the diet switch, and compared to ND-fed group (*n* = 3 mice for ND group, *n* = 6 mice for RES, and RES + CD8T depletion groups). **b** Representative CD4/CD8α staining of CD45^+^TCRβ^+^ NK1.1^**−**^ gated liver MNCs. **c** Body weight. **d** Wet liver weight. **e** Serum ALT levels. **f** Representative photomicrographs of Masson trichrome staining of liver sections. Scale bars: 200 µm. **g** Representative photomicrographs (left) and positive area (right) of Sirius Red staining of liver sections. Scale bars: 200 µm. **h** Hydroxyproline levels. **i** Fibrosis associated genes (*Col1a1, Col1a2, Acta2, Timp1, Desmin, Spp1)* mRNA levels. **j** (left) Representative fluorescent photomicrographs of liver sections stained with 4′,6-diamidino-2-phenylindole (DAPI: blue) and anti-desmin Ab (red). Scale bars: 200 µm. (right) HSCs count in unit area of liver section. **k** Number of CD45^+^CD11b^+^CD11c^**−**^ macrophages in CD45^+^ liver MNCs. Data are presented as mean ± SEM. One-way ANOVA with Tukey’s multiple comparisons post-hoc test was applied. Source data are provided as a Source data file.

To elucidate the role of CD8^+^ T cells in NASH resolution, we treated mice with either anti-CD8α killing antibody or the isotype control during the recovery phase (Fig. 2a). Results confirmed that the CD8^+^ T cell subset was efficiently depleted 5 weeks following initiation of resolution (Fig. 2b). Although depletion of CD8^+^ T cells did not affect body weight or liver weight (Fig. 2c, d), recovery from liver inflammation and fibrosis was prevented in the absence of CD8^+^ T cells (Fig. 2e–i), while not prevented in mice depleted of natural killer (NK) cells and NKT cells (Supplementary Fig. 2a–d), other known antifibrotic cell subsets^19,20^, nor in mice depleted of conventional dendritic cells (DCs) or plasmacytoid DCs (Supplementary Fig. 2e–h), a part of which express CD8. Notably, depletion of CD8^+^ T cells increased hepatic stellate cells (HSCs) (Fig. 2j), while the number of macrophages remained unchanged (Fig. 2k). These data collectively support that CD8^+^ T cells as a whole play a substantial role in resolving the fibrosis.

**Fig. 3:**
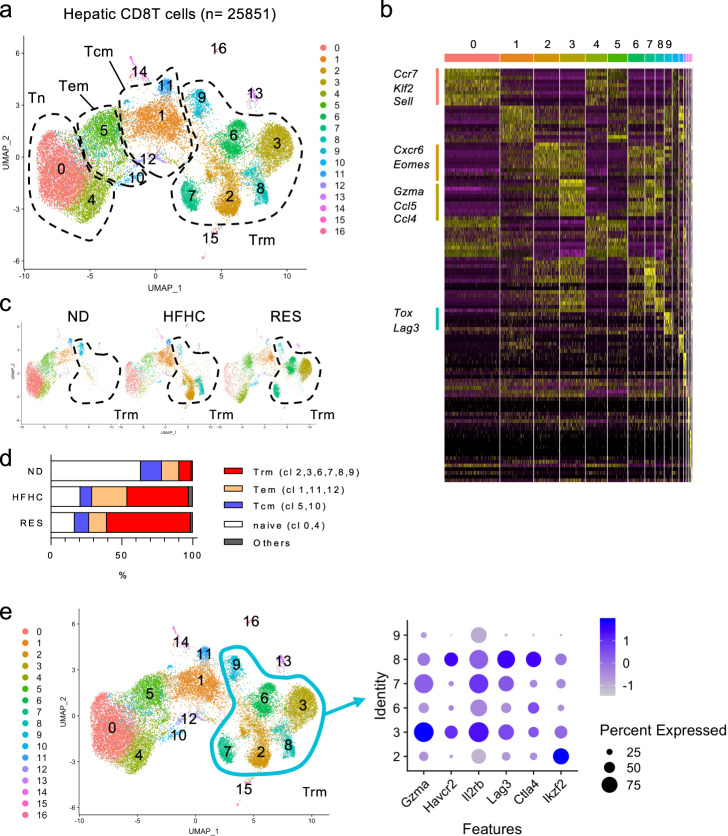
Landscape of CD8^+^ T cells in NASH and NASH resolution livers. **a**–**d** ScRNA-seq analysis of liver CD8 T cells. **a** UMAP projection of combined liver CD8^+^ T cells (*n* = 25,851) derived from ND, HFHC, and RES (5 weeks) mice, showing the formation of 17 main clusters represented by different colors. The functional description of each cluster is determined by the gene expression characteristics of each. **b** Heat map of 17 CD8^+^ T cell clusters with top 10 unique signature genes. **c** UMAP projection of liver CD8^+^ T cells derived from ND (left), HFHC (middle), and RES (right) mice. **d** Fractions of five clusters (naïve, Tcm, Tem, Trm, and others) in CD8^+^ T cells. **e** Dot plot visualizing the expression of representative genes in the Trm clusters (cluster 2, 3, 6–9). The color represents the average expression level, and the circle size represents the proportion of cells expressing each gene. Source data are provided as a Source data file.

**Fig. 4:**
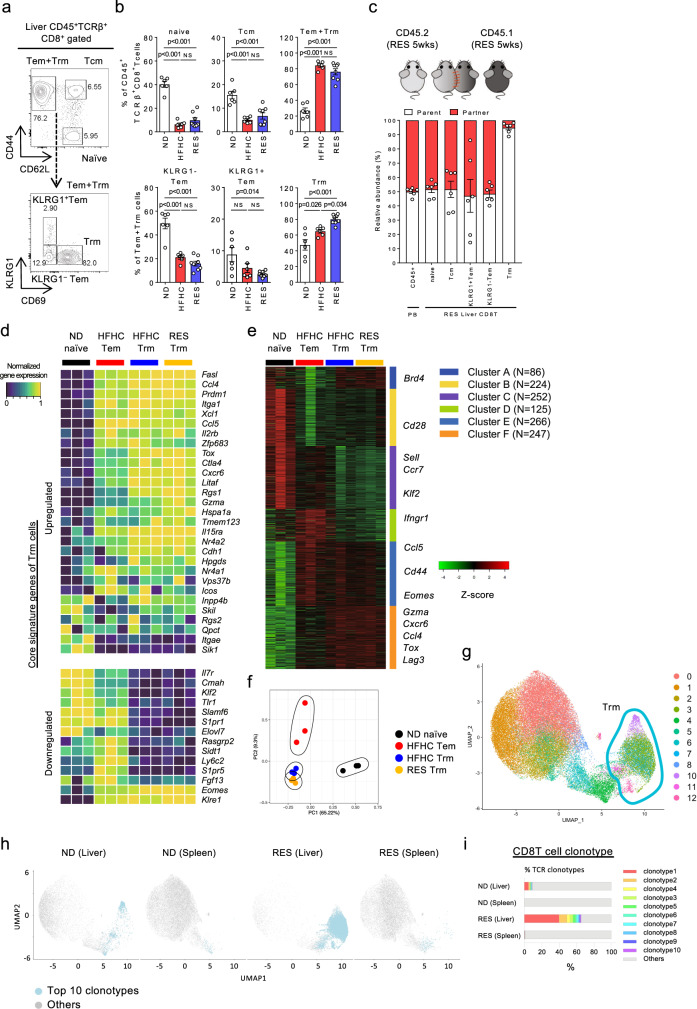
Characteristics of CD8^+^ Trm cells in NASH and NASH resolution livers. **a** Representative CD44/CD62L staining of CD45^+^TCRβ^+^NK1.1^**−**^CD8α^+^ gated liver MNCs (upper) and KLRG1/CD69 staining of CD45^+^TCRβ^+^NK1.1^**−**^CD8α^+^CD44^+^CD62L^**−**^ gated (Tem + Trm) liver MNCs (lower) of RES (5 weeks) mice. **b** Frequency of each CD8^+^ T cell subset in CD45^+^ liver MNCs derived from the indicated groups of mice (*n* = 6 mice for ND and HFHC groups, and *n* = 8 mice for RES group). Data are presented as mean ± SEM. One-way ANOVA with Tukey’s multiple comparisons post-hoc test was applied. **c** Relative abundance of partner or parent cells in the blood and liver MNCs of mice two weeks following the parabiosis surgery between Ly5.2^+^ RES (5 weeks) mice and Ly5.1^+^ RES (5 weeks) mice (*n* = 6 pairs). Data are presented as mean ± SEM. **d**–**f** Bulk RNA-seq analysis of isolated liver naïve CD8^+^ T cells from ND mice (ND naïve; *n* = 3), CD8^+^ Tem cells from HFHC mice (HFHC Tem; *n* = 3), CD8^+^ Trm cells from HFHC mice (HFHC Trm; *n* = 3), and CD8^+^ Trm cells from RES (5 weeks) mice (RES Trm; *n* = 3). **d** Heat map of key upregulated (upper) or down-regulated (lower) genes characteristic of Trm. **e** K-means clustering analysis. Most variable 1200 genes were clustered to six groups. Featured genes determined by scRNA-seq were shown. **f** PCA analysis plot of top 1000 genes. **g**–**i** ScTCR-seq analysis of combined liver or spleen CD8 T cells derived from ND and RES (5 weeks) mice. **g** UMAP projection of combined liver and spleen CD8^+^ T cells (*n* = 53,659) derived from ND(Liver), ND (Spleen), RES (Liver), and RES (Spleen) mice. Green circle highlights clusters of Trm cells. **h** UMAP projection and **i** percentage of top 10 and other TCR clonotypes in each cell subset. Source data are provided as a Source data file.

To reveal the transcriptome profiling-based subtypes of CD8^+^ T cells present during the NASH resolution phase, we performed single-cell RNA sequencing (scRNA-seq) analysis using sorted CD8^+^ T cells from the livers of ND, HFHC, and RES mice, respectively. First, a total of 25,851 cells were clustered into 17 clusters based on the gene signatures (Fig. 3a, b). Of interest, the composition of clusters and corresponding gene expression of sorted CD8^+^ T cells were clearly distinct from each other (Fig. 3c and Supplementary Fig. 3). Based on the combination of *Cd44, Sell*, and *S1pr1* genes expression, the clusters were divided into four groups composed of *Cd44*^−^*Sell*^+^*S1pr1*^+^ naïve T cells (cluster 0, 4) expressing *Ccr7, Klf2, and Sell*, *Cd44*^+^*Sell*^+^*S1pr1*^+^ central memory T cells (Tcm; cluster 5, 10), *Cd44*^+^*Sell*^**−**^*S1pr1*^+^ effector memory T cells (Tem; cluster 1, 11, 12), *Cd44*^+^*Sell*^**−**^*S1pr1*^**−**^ resident memory T cells (Trm; cluster 2, 3, 6, 7, 8, 9) expressing *Cxcr6, Eomes, Gzma, Ccl5, Ccl4, Tox, and Lag3*, and others (cluster 13–16), demonstrating that >60% of ND CD8^+^ T cells were naïve T cells, while RES CD8^+^ T cells were abundant in Trm cells with fewer naïve T cells (Fig. 3d). Of the Trm subsets, cluster 3 (characteristic in RES CD8 T cells) featured *Gzma* and *Il2rb*, while cluster 8 (characteristic in HFHC CD8 T cells) contained *Lag3* and *Ctla4* (Fig. 3e). These findings were further validated by flowcytometry demonstrating that CD44^+^CD62L^**−**^CD69^+^CD8^+^ T cells, characteristic of Trm were significantly increased in the liver of RES mice (Fig. 4a, b). A large proportion of liver CD8^+^ Trm cells expressed CXCR3 and CXCR6, while few CD103 expression was shown (Supplementary Fig. 4). Tissue residency of these cells with lower circulating potential was confirmed in parabiosis mice in which blood circulation was shared between Ly5.1 and Ly5.2 RES mice (Fig. 4c). Furthermore, bulk RNA-seq analysis of isolated cells (ND naïve, HFHC Tem, HFHC Trm, and RES Trm) revealed that both Trm subsets from HFHC mice and RES mice expressed core signature genes of Trm^21–24^ with exceptions including *Itgae* (CD103) and *Eomes* (Fig. 4d). Six clusters were classified based on the gene expression profiles by K-means clustering, demonstrating that featured genes in scRNA-seq analysis were consistently expressed in each cell subset (Fig. 4e). These four cell types were distinct from each other in global transcriptome (Fig. 4f), while GO analysis demonstrated upregulation of genes related to chemotaxis and inflammatory pathways in Trm (Supplementary Fig. 5a). Moreover, genes associated with cytotoxic mediators including *Gzma, Gzmb, Gzmk, Fasl*, and chemotaxis mediators including *Ccl3* and *Ccl4* were found to be highly expressed in RES Trm compared to HFHC Tem (Supplementary Fig. 5b). Meanwhile, RES Trm showed higher expression of *Gzma* and *Serpin* families, while showed lower expression of *Spp1, Ltb, and Tgfb3*, compared to HFHC Trm (Supplementary Fig. 5c). Of the genes related to chemotaxis and inflammatory pathways, we confirmed that the absolute transcript levels of *CCl3, CCl4, CCl5, Prf1, Fasl, Ifng*, and *Gzma* was abundant in Trm, while previously reported fibrinolysis-associated genes including *Tnfsf10, Mmp9, Mmp12, Mmp13* were not expressed in Trm (Supplementary Fig. 5d). TCR repertoire analysis suggested that multiple antigenicity could predispose the differentiation of CD8^+^ Trm cells during NASH progression/resolution (Supplementary Fig. 6a). Meanwhile, scTCR-seq analysis further revealed the clonal enrichment mainly in the *Cd44*^+^*Sell*^**−**^*S1pr1*^**−**^ liver Trm fractions (Fig. 4g, h and Supplementary Fig. 6b). In addition, TCR clonotypes enriched in liver CD8^+^ Trm cells of RES mice were expanded compared to those in liver CD8^+^ T cells of ND mice, and rarely detected in spleen CD8^+^ T cells (Fig. 4h, i and Supplementary Fig. 6b, c), suggesting that these cells originated from the liver rather than recruited from the spleen. These results collectively demonstrate that unique CD8^+^ Trm cells are present in NASH resolution livers, suggesting a specific role in resolving liver fibrosis.

To elucidate the comprehensive role of CD8^+^ T cells in NASH resolution, we applied an methionine choline deficient (MCD) diet model, another widely used murine NASH model characterized by the rapid progression, followed by a switch to ND (Supplementary Fig. 7a). Liver inflammation and liver fibrosis, as assessed by serum ALT levels, hepatic *Col1a1* gene expression, and hydroxyproline levels, were restored to the original level by day 7 after the diet switch (Supplementary Fig. 7b–d). Consistently, the proportion of CD8^+^ Trm cells was significantly increased in the liver of fibrosis-resolved mice compared to that in the liver of MCD mice (Supplementary Fig. 7e), while ablation of CD8^+^ T cells negated the resolution of fibrosis (Supplementary Fig. 7f–i), reinforcing the role of CD8^+^ T cells in NASH resolution.

### Resolution phase CD8^+^ T cell transfer inhibits NASH development

**Fig. 5:**
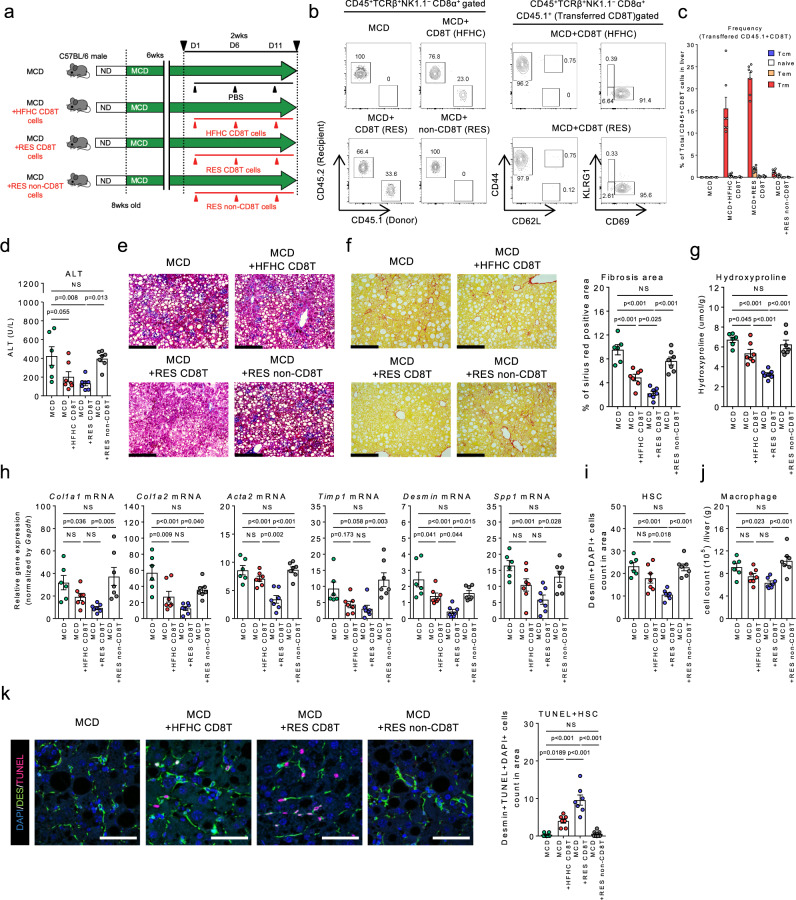
Direct antifibrotic effect of CD8^+^ Trm cells in NASH progression. **a** Study design: male C57BL/6 (Ly5.2) mice were fed a MCD diet for 8 weeks. Mice were intravenously injected with either PBS, liver CD8T cells isolated from Ly5.1 HFHC mice or Ly5.1RES (5 weeks) mice, or liver non-CD8T cells isolated from RES mice (2 × 10^6^ cells each) on day 1, 6, and 11 starting from week six (*n* = 6 mice for MCD group, and *n* = 7 mice for the other groups). **b** Representative CD45.2/CD45.1 staining of CD45^+^TCRβ^+^NK1.1^**−**^CD8α^+^ gated liver MNCs (left). Representative CD44/CD62L (center) and KLRG1/CD69 (right) staining of CD45^+^TCRβ^+^NK1.1^**−**^CD8α^+^ CD45.1^+^ (Transferred CD8T) gated liver MNCs. **c** Frequency of the transferred CD8^+^ T cell subset in CD45^+^ liver MNCs of the indicated groups of mice. **d** Serum ALT levels. **e** Representative photomicrographs of Masson trichrome staining of liver sections. Scale bars: 200 µm. **f** Representative photomicrographs (left) and positive area (right) of Sirius Red staining of liver sections. Scale bars: 200 µm. **g** Hydroxyproline levels. **h** Fibrosis associated genes (*Col1a1, Col1a2, Acta2, Timp1, Desmin, Spp1)* mRNA levels. **i** HSCs count in unit area of liver section. **j** Number of CD45^+^CD11b^+^ CD11c^**−**^ macrophages in CD45^+^ liver MNCs. **k** (left) Representative fluorescent photomicrographs of liver sections stained with DAPI (blue), anti-desmin Ab (green), and TUNEL (pink). Scale bars: 50 µm. (right) Count of TUNEL positive HSCs in unit area of liver section. Data are presented as mean ± SEM. One-way ANOVA with Tukey’s multiple comparisons post-hoc test was applied. Source data are provided as a Source data file.

To further determine the specific role of CD8^+^ T cells in NASH progression, we isolated CD8T cells and non-CD8T cells from HFHC mice or RES mice (Ly5.1). Each cell subset was then continuously injected to MCD diet-fed Ly5.2 mice in which the effect of cell transfer can be efficiently evaluated for its rapid fibrosis progression (Fig. 5a). While CD8^+^ T cells efficiently migrated in the recipient livers, a large proportion of the accumulated CD8^+^ T cells in the livers of mice transplanted with CD8T cells was CD44^+^CD62L^**−**^CD69^+^CD8^+^ Trm (Fig. 5b, c). Although mice transplanted with HFHC CD8T cells demonstrated a modest reduction in liver inflammation and fibrosis, RES CD8T cells achieved a significant superior effect (Fig. 5d–h) partly by inducing cell death of HSCs with less contribution of macrophages (Fig. 5i–k), underpinning a role for RES CD8^+^ Trm cells in suppressing NASH progression, in addition to their role in NASH resolution. More specifically, we confirmed that transfer of sorted CD8^+^ Trm cells derived from RES mice directly protected from MCD diet-induced fibrosis progression (Supplementary Fig. 8a–e).

### IL-15 maintains liver CD8^+^ Trm cell population

**Fig. 6:**
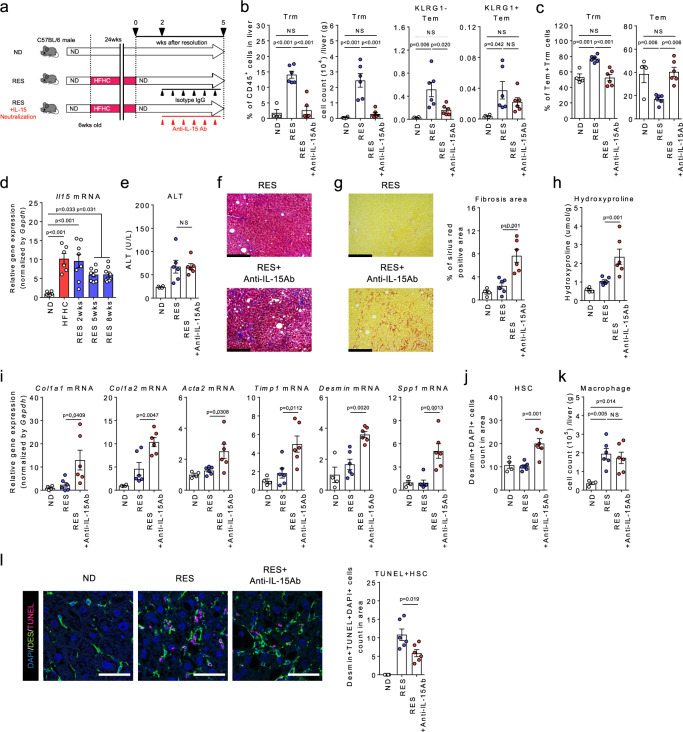
Roles for IL-15 signaling in the maintenance of CD8^+^ Trm cells in NASH resolution. **a** Study design: resolution induced mice were intraperitoneally injected with anti-IL-15 antibody (RES + IL-15 neutralization group) or the isotype control (RES group) once every 3 days starting from 2 weeks following the diet switch to 5 weeks, and compared to the ND-fed mice (*n* = 4 mice for ND group, and *n* = 6 mice for RES and RES + IL-15 neutralization groups). **b** Proportion and number of CD8^+^ Trm cells and number of KLRG1^**−**^ CD8^+^ Tem and KLRG1^+^ CD8^+^ Tem in the liver CD45^+^ MNCs. **c** Proportion of CD8^+^ Trm cells (left) and Tem cells (left) in the combined population. **d** *Il15* mRNA levels in the whole liver (*n* = 6 mice for ND and HFHC group, and *n* = 8 mice for RES groups). **e** Serum ALT levels. **f** Representative photomicrographs of Masson trichrome staining of liver sections. Scale bars: 200 µm. **g** Representative photomicrographs (left) and positive area (right) of Sirius Red staining of liver sections. Scale bars: 200 µm. **h** Hydroxyproline levels. **i** Fibrosis associated genes (*Col1a1, Col1a2, Acta2, Timp1, Desmin, Spp1)* mRNA levels. **j** HSCs count in unit area of liver section. **k** Number of CD45^+^CD11b^+^CD11c^**−**^ macrophages in CD45^+^ liver MNCs. **l** (left) Representative fluorescent photomicrographs of liver sections stained with DAPI (blue), anti-desmin Ab (green), and TUNEL (pink). Scale bars: 50 µm. (right) Count of TUNEL positive HSCs in unit area of liver section. Data are presented as mean ± SEM. One-way ANOVA with Tukey’s multiple comparisons post-hoc test was applied. Source data are provided as a Source data file.

Given that Trm has a potential to suppress liver fibrosis, we determined whether depletion of Trm exaggerate NASH progression. Since RES Trm cells highly expresses IL-15 receptor genes (*Il15ra*, *Il2rb*) (Figs. 3e and 4d), we antagonized IL-15 signaling to investigate the role of IL-15 in the maintenance of CD8^+^ Trm cells and the subsequent effect on NASH resolution (Fig. 6a). As expected, IL-15 neutralization resulted in a dramatic reduction in the number and proportion of CD8^+^ Trm, but not Tem, in the liver (Fig. 6b, c). Consistently, IL-15 gene expression was upregulated in HFHC livers, which was maintained throughout the resolution phase of NASH (Fig. 6d). Consequently, recovery from the established fibrosis with increased HSCs death was significantly inhibited, while little effect was observed in liver inflammation, as determined by serum ALT levels (Fig. 6e–l). These results clearly suggest that IL-15 is crucial for the maintenance of CD8^+^ Trm cells and is directly involved in NASH resolution. Similarly, blockade of CXCR3, another retention marker of Trm in the liver, efficiently eliminated Trm and Tem cells from the resolution livers and partially inhibited fibrosis resolution (Supplementary Fig. 9a–e).

### Interplay between CD8^+^ Trm cells and HSCs contributes to NASH resolution in a CCR5-dependent manner

**Fig. 7:**
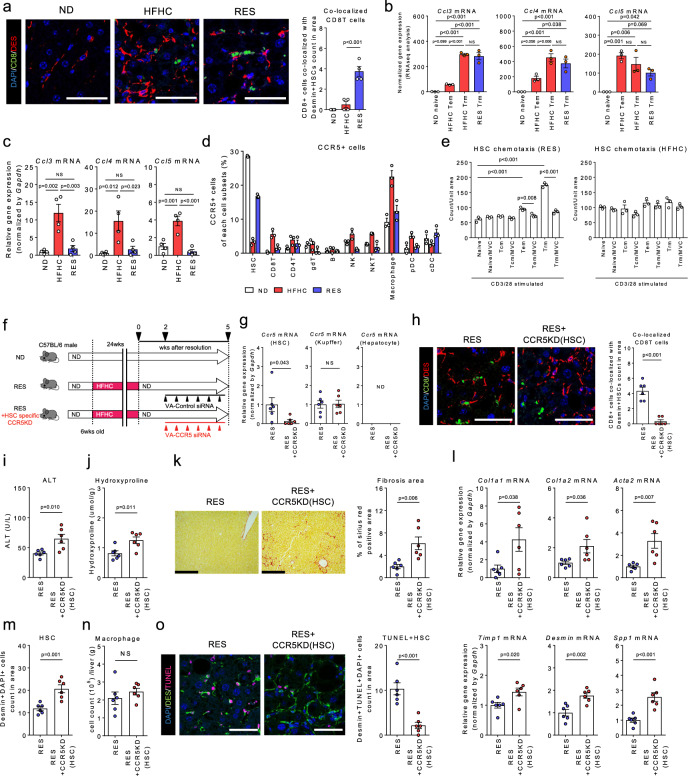
CD8^+^ Trm cells target HSCs in a CCR5-dependent manner in NASH resolution. **a** (Left) Representative fluorescent photomicrographs of liver sections of ND, HFHC, and RES (3 weeks) mice stained with 4′,6-diamidino-2-phenylindole (DAPI: blue), anti-CD8 Ab (green), and anti-desmin Ab (red). Insets show higher magnification. Scale bars: 50 µm. (Right) Count of CD8^+^ T cells co-localized with HSCs in unit area of liver section　(*n* = 4 mice per group). **b**, **c** *Ccl3, Ccl4, and Ccl5* mRNA levels in sorted CD8 T cell subsets (**b**) (*n* = 3 mice per group), and isolated hepatocytes (**c**) (*n* = 4 mice per group). **d** Frequency of CCR5^+^ cells in CD45^+^ liver MNCs and HSCs of the indicated groups (*n* = 3 mice per group). **e** Cell numbers of migrated HSCs in lower compartment containing supernatants of CD3/28-stimulated CD8^+^ T cell subset with or without CCR5 inhibitor, Marovenic (MVC) (left; HSCs and CD8^+^ T cells isolated from RES mice, right; isolated from HFHC mice, *n* = 3 biologically independent samples per group). **f** Study design: RES mice were intravenously treated with vitamin A-control siRNA liposomes (RES group) or vitamin A-*Ccr5* siRNA liposomes (RES + HSC specific CCR5KD group) twice per week starting from 2 weeks following the diet switch to 5 weeks (*n* = 6 mice per group). **g** *Ccr5* mRNA levels in isolated HSCs, Kupffer cells, and hepatocytes of the indicated groups. **h** (left) Representative fluorescent photomicrographs of liver sections stained with DAPI (blue), anti-desmin Ab (red), and anti-CD8 Ab (green). Scale bars: 50 µm. (right) Count of CD8^+^ T cells co-localized with HSCs in unit area of liver section. **i** Serum ALT levels. **j** Hydroxyproline levels. **k** Representative photomicrographs (left) and positive area (right) of Sirius Red staining of liver sections. Scale bars: 200 µm. **l** Fibrosis associated genes (*Col1a1, Col1a2, Acta2, Timp1, Desmin, Spp1)* mRNA levels. **m** HSCs count in unit area of liver section. **n** Number of CD45^+^CD11b^+^CD11c^**−**^ macrophages in CD45^+^ liver MNCs. **o** (left) Representative fluorescent photomicrographs of liver sections stained with DAPI (blue), anti-desmin Ab (green), and TUNEL (pink). Scale bars: 50 µm. (right) Count of TUNEL positive HSCs in unit area of liver section. Data are presented as mean ± SEM. One-way ANOVA with Tukey’s multiple comparisons post-hoc test (**a**–**c**, **e**) or two-sided unpaired Student’s *t* test (**g**–**o**) was applied. Source data are provided as a Source data file.

We next sought to investigate the antifibrotic roles of CD8^+^ T cells during development and resolution of NASH. To this end, we explored the chemotactic factors that could facilitate close proximity between CD8^+^ T cells and HSCs in the liver of RES mice (Fig. 7a). RNA-seq analysis revealed robust upregulation of *CCl3, CCl4, and CCl5* genes in both HFHC and RES CD8^+^ Trm (Fig. 7b). Of note, these genes were also upregulated in hepatocytes during the fibrosis phase, however, were regressed in the resolution phase (Fig. 7c). Regarding the cells expressing the corresponding receptor, CCR5, in the liver, HSCs exhibited the highest expression in the steady state, while its expression was lost at the peak of fibrosis and regained during the resolution phase. Conversely, macrophages showed the highest expression at the peak of fibrosis phase (Fig. 7d). Moreover, in vitro, HSCs demonstrated CCR5-dependent chemotaxis to RES CD8^+^ Trm, while that between HSCs and HFHC CD8^+^ Trm was less evident (Fig. 7e), suggesting that the interplay was regulated according to the fibrous niche. To further confirm the requirement for CCR5 in fibrosis resolution, we generated HSC-specific CCR5 deficient mice by administrating vitamin A-CCR5 siRNA liposomes during the NASH resolution phase (Fig. 7f). We confirmed that CCR5 gene expression was specifically abolished in HSCs (Fig. 7g). Notably, the proximity between CD8^+^ Trm cells and HSCs was less prominent by HSC-specific CCR5 knockdown (Fig. 7h). Consequently, the recovery from both inflammation and fibrosis with increased HSCs death was significantly attenuated (Fig. 7i–o). These results collectively reinforced the substantial role of CCR5-expressing HSCs in NASH resolution.

### Trm cells induce HSC apoptosis via FasL-Fas during NASH resolution

**Fig. 8:**
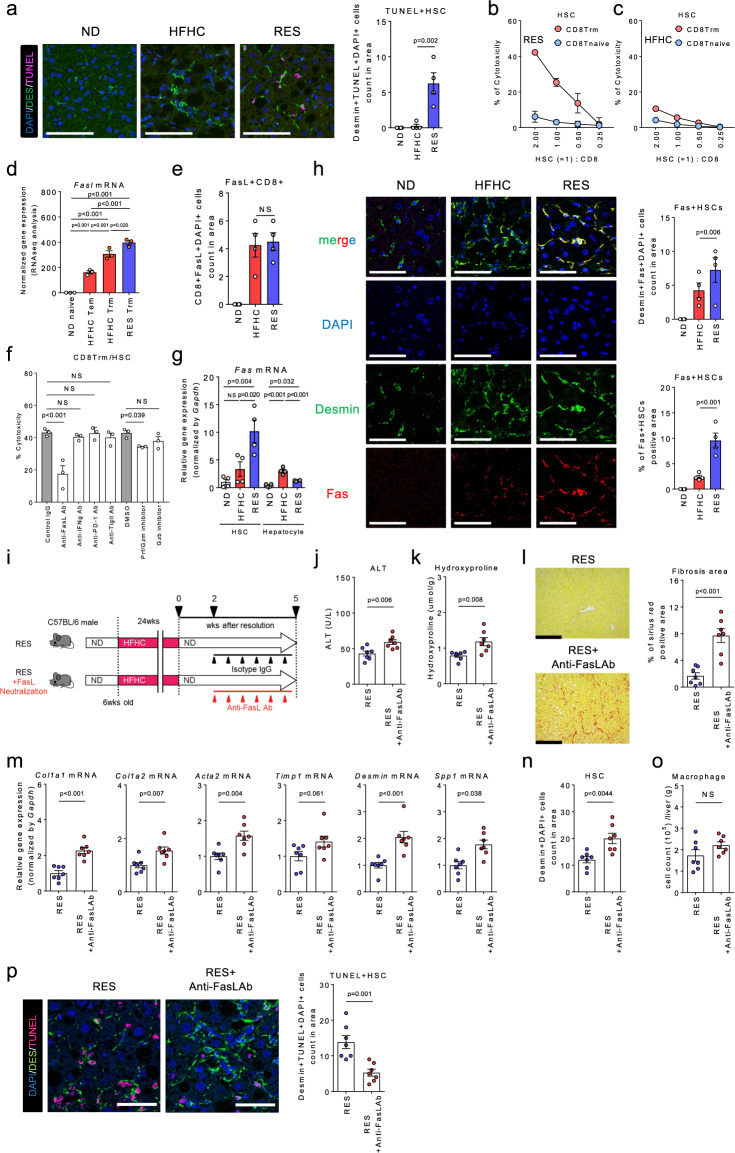
CD8^+^ Trm cells induce HSCs apoptosis in a FasL dependent manner in NASH resolution. **a** (left) Representative fluorescent photomicrographs of liver sections from ND, HFHC, and RES (3 weeks) mice stained with anti-desmin Ab (green) and TUNEL (pink). Scale bars: 50 µm. (right) Count of TUNEL positive HSCs in unit area of liver section (*n* = 4 mice per group). **b**, **c** Percent cytotoxicity of HSCs by CD8 Trm (red) or CD8 T naive (blue) isolated from RES 5 weeks mice (**b**) or HFHC mice (**c**) (*n* = 3 biologically independent samples per group). **d** *Fasl* mRNA levels in sorted CD8^+^ T cell subsets (*n* = 3 mice per group). **e** Count of FasL positive CD8^+^ T cells in unit area of liver section (*n* = 4 mice per group). **f** Percent cytotoxicity of HSCs co-cultured with CD8^+^ Trm cells in the presence of the indicated apoptosis inhibitors for 8 h (*n* = 3 biologically independent samples per group). **g** *Fas* mRNA levels in isolated HSCs and hepatocytes of the indicated mice (*n* = 4 mice per group). **h** (left) Representative fluorescent photomicrographs of liver sections stained with DAPI (blue), anti-desmin Ab (green), and anti-Fas (red). Scale bars: 50 µm. (right upper) Count of Fas positive HSCs in unit area of liver section (*n* = 4 mice per group). (right lower) Positive area of Fas positive HSCs in unit area of liver section. **i** Study design: resolution induced mice were intraperitoneally injected with anti-FasL antibody (RES + FasL neutralization group) or the isotype control (RES group) once every 3 days for 3 weeks starting at week 2 following diet switch (*n* = 7 mice per group). **j** Serum ALT levels. **k** Hydroxyproline levels. **l** Representative photomicrographs (left) and positive area (right) of Sirius Red staining of liver sections. Scale bars: 200 µm. **m** Fibrosis associated genes (*Col1a1, Col1a2, Acta2, Timp1, Desmin, Spp1)* mRNA levels. **n** HSCs count in unit area of liver section. **o** Number of CD45^+^CD11b^+^ CD11c^**−**^ macrophages in CD45^+^ liver MNCs. **p** (left) Representative fluorescent photomicrographs of liver sections stained with DAPI (blue), anti-desmin Ab (green), and TUNEL (pink). Scale bars: 50 µm. (right) Count of TUNEL positive HSCs in unit area of liver section. Data are presented as mean ± SEM. One-way ANOVA with Tukey’s multiple comparisons post-hoc test (**a**, **d**–**h**) or two-sided unpaired Student’s *t* test (**j**–**p**) was applied. Source data are provided as a Source data file.

Regarding the interplay between CD8^+^ Trm cells and HSCs, immunohistochemical analysis revealed that HSCs in RES mice exhibited apoptotic characteristics of cell death confirmed using TUNEL (Fig. 8a) and cleaved Caspase 3 staining (Supplementary Fig. 10a). Furthermore, RES CD8^+^ Trm cells demonstrated a direct cytotoxic effect on HSCs in vitro, while apoptosis was induced to a lesser extent by RES CD8^+^ naïve T cells or HFHC CD8^+^ Trm cells (Fig. 8b, c). Of note, the apoptosis-inducing effect was not evident in hepatocytes (Supplementary Fig. 10b, c), suggesting a HSCs-specific action. Genes profile from transcriptome and immunohistochemical analyses illustrated the associated molecular mechanism underlaying the HSC-targeted cytotoxicity (Fig. 8d, e and Supplementary Fig. 10d–g). Of the candidate apoptosis-inducing molecules, we confirmed that FasL as well as Perforin/Granzyme play substantial roles in the cytotoxic effect elicited by CD8^+^ Trm on HSCs (Fig. 8f). Consistently, *Fas* gene and protein expression was specifically upregulated in the HSCs derived from RES mice (Fig. 8g, h), suggesting a phase- and cell- dependent susceptibility to cytotoxicity. Finally, we confirmed that treatment with anti-FasL Ab during the resolution phase partially prevented the regression from NASH (Fig. 8i–o) and decreased apoptotic HSCs (Fig. 8p). These results collectively reveal a potential mechanism by which deactivation of HSCs by CD8^+^ Trm cells is regulated during NASH resolution.

### CD69^+^CD8^+^ Trm cell number in human liver is associated with NASH progression

**Fig. 9:**
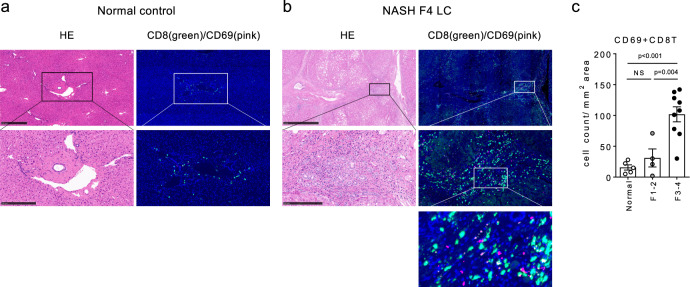
Abundance of CD69^+^CD8^+^ Trm cells in human advanced NASH livers. **a**, **b** Representative (left) H&E staining and (right) immunofluorescent staining with anti-CD8α Ab and anti-CD69 Abs of resected liver tissues from normal control (**a**) and NASH LC (F4) patient (**b**). Middle and lower panels show each upper image at a higher magnification. Scale bars: 500 µm (normal control) or 1 mm (NASH F4) in the top panel, 250 µm in the middle panel. **c** Number of CD69^+^ and CD8^+^ co-stained cells in the liver of indicated groups of patients (*n* = 5 for normal control, *n* = 4 for F1-2, and *n* = 9 for F3-4). Data are presented as mean ± SEM. One-way ANOVA with Tukey’s multiple comparisons post-hoc test was applied. Source data are provided as a Source data file.

Finally, we determined whether our findings could be translated to human NASH patients. For this, liver samples from 13 NASH patients were evaluated (Supplementary Table 1). Immunohistochemical staining demonstrated that a proportion of CD8^+^ T cells accumulated in the portal area co-expressed CD69, while the number of co-expressing cells was significantly increased as liver fibrosis progressed (Fig. 9a–c), supporting a potential role for CD69^+^CD8^+^ Trm cells in regulating NASH development.

## Discussion

Recent advance in the field of adaptive immune system have elucidated the role of CD8^+^ Trm cells in anti-tumor immunity^25,26^ or inflammatory autoimmune diseases^27^ in addition to their original role in infectious diseases^28,29^. Our study provides a previously unappreciated role of liver CD8^+^ Trm cells in fibrosis resolution. We here demonstrated that CD8^+^ Trm cells promote this process through the mechanism of CCR5-dependent chemoattraction and direct cytotoxic effect on HSCs.

The principal hallmark of bona fide Trm is their long-term persistence in non-lymphoid tissues without recirculation in the blood due to a transcriptional loss of *S1pr1* and *Klf2*^21,30^. Differentiation of Trm is controlled by complex combinations of several transcription factors in each tissue, and the requirement differs between mice and humans^31^. Of the transcription factors responsible for Trm differentiation, hepatic CD8^+^ Trm cells from HFHC and RES mice constitutively exhibit a Hobit^int^Blimp-1^high^Eomes^high^ phenotype. As downregulation of Eomes is required for CD103^+^CD8^+^ Trm development via TGF-β signaling^32^, insufficient local TGF-β signaling may result in sustained high Eomes expression and an abundance of CD103^**−**^CD69^+^CD8^+^ Trm cells in the liver, consistent with previous reports^28,33^. Furthermore, it has been reported that CD103 may not be fully responsible for the retention of intrahepatic Trm cells in mice^28,34^, suggesting another molecular mechanism underlying the global localization of CD8^+^ Trm cells in the liver sinusoids. In this regard, our current results, along with the previous findings^28,35^, suggest that chemokine receptor CXCR3 and CXCR6 may be required for the localization. Regarding the maintenance of CD8^+^ Trm cells in the liver, we found that *Il2rb*, a subunit of IL-15 receptor, is highly expressed in RES CD8^+^ Trm cells, whereas its neutralization during NASH resolution diminishes CD8^+^ Trm cells and prevents fibrosis resolution, underscoring the requirement for IL-15 in the maintenance of CD8^+^ Trm cells during NASH resolution. Besides cytokine signaling, differentiation of CD8^+^ Trm cells can be directed by local antigens presentation and TCR engagement. The TCR repertoire analysis suggested that multiple antigenic stimulation, including from gut-derived or intrinsic molecules, may contribute to the development of CD8^+^ Trm cells in NASH development and resolution. Enrichment of TCR clonotypes in RES livers, which is distinct from that in spleens, further suggests that the local antigenic stimulation can induce the development and expansion of CD8^+^ Trm cells during NASH resolution. Further studies are warranted to identify the specific antigens that contribute to this development in the liver.

Hepatic Trm cells recognize and target infected hepatocytes in viral and parasite infection^28,36^, however, it is unclear whether and how Trm cells interact with other non-parenchymal cells in the liver sinusoid. One key finding of our study is the CCR5-dependent interplay between CD8^+^ Trm cells and HSCs in NASH resolution. In contrast, during liver fibrosis progression, CCR5 plays a profibrotic role in the arc of hepatocytes, macrophages, and HSCs^37,38^. The dynamics of CCR5 ligand gene expression in hepatocytes during fibrosis progression/resolution support that the inflammatory niche regulates the complex chemokine-dependent cellular network and the subsequent fate of HSCs. Of the proposed fate of activated HSCs^9,39,40^, we demonstrated induction of FasL/Fas-dependent apoptosis by CD8^+^ Trm cells during fibrosis resolution. Notably, the cytotoxic effect of CD8^+^ Trm cells was regulated by the fibrosis phase as well as by the targeted cells. Meanwhile, potent antifibrotic effects of RES CD8^+^ Trm cells demonstrated in ongoing fibrosis by adoptive cell transfer suggests an alternative fibrinolytic mechanism aside from the niche-restricted interaction with HSCs. Toward this point, transcriptome analysis revealed several candidate genes responsible for the superior antifibrotic effect of RES CD8^+^ Trm over HFHC CD8^+^ Trm, including upregulation of *Spp1* (Osteopontin) and *Ltb* (Lymphotoxin β) in HFHC CD8^+^ Trm, and upregulation of *Gzma* (Granzyme A) in RES CD8^+^ Trm. Of these, granzyme A has been reported to disrupt the extracellular matrix by cleaving type 4 collagen and fibronectin^41^, in addition to the intracellular cytotoxic effect; thus, it may directly contribute to fibrosis resolution. However, further studies are required to clarify its actual contribution.

The current study collectively provides additional insights into the liver protective function of CD8^+^ Trm cells in NASH pathogenesis. As a larger number of CD69^+^ CD8^+^ Trm cells were observed in the liver of patients with advanced NASH, attempts to manipulate their number and/or function may serve as a potential therapeutic option for NASH in the future.

## Methods

### Animals

C57BL/6 mice were purchased from Japan CLEA. CD11c^dtr/dtr^ mice on a C57BL/6 background were provided from Dr. Kenya Honda (Keio University). Siglech^dtr/dtr^ mice on a C57BL/6 background were established previously^42^. C57BL/6-Ly5.1 mice were obtained from Taconic Laboratory. All mice were maintained under specific pathogen–free (SPF) conditions with a 12-h light/dark cycle, at a temperature of 22–25 °C and a relative humidity of 45–55% in the Animal Care Facility of Keio University School of Medicine. All animal experiments have complied with all relevant ethical regulation approved by Animal Ethics Committee of Keio University.

### NASH resolution study design

To induce NASH, male C57BL/6 mice (6 weeks old) were fed a 60% high-fat 1% high-cholesterol (HFHC) diet purchased from Japan CLEA (Tokyo, Japan) for 24 weeks^43^, or fed a MCD diet purchased from Research Diets Inc. (NJ, USA) for seven weeks. Mice were then switched from the NASH-inducing diets to a normal diet (CE2; Japan CLEA) to induce NASH resolution after randomization by the ALT value and body weight. The mice were fed a normal diet for either 2–8 weeks (HFHC diet) or 1 week (MCD diet).

### Isolation of liver immune cells, HSCs, and hepatocytes

Liver mononuclear cells (MNCs) were separated from the liver as described previously with slight modifications^44^. Briefly, livers were perfused through the postcaval vein with HBSS (Nacalai Tesque, Kyoto, Japan) and then minced and homogenized by gentle MACS (Miltenyii biotec japan, Tokyo, Japan). The suspensions were passed through 100 μm nylon mesh, centrifuged at 50 g for 5 min, and the supernatant was washed once. Cells were suspended in 40% Percoll and overlaid on a 75% Percoll fraction. Percoll gradient separation was performed by centrifugation at 840×*g* for 20 min at room temperature. MNCs were collected at the interface, washed, and resuspended in FACS buffer or RPMI-1640 medium (Sigma-Aldrich, MO, USA) supplemented with 10% fetal bovine serum and 1% penicillin/streptomycin (Nacalai Tesque). Liver HSCs and hepatocytes were isolated from mice. In brief, the method based on in situ protease/Collagenase perfusion was used. In all, 25 mL of PBS (Nacalai Tesque), 20 mL of 2 mg/mL protease (Sigma-Aldrich) solution in DMEM (Nacalai Tesque), and 40 mL of 0.5 mg/mL collagenase (Sigma-Aldrich) in DMEM solution were pre-warmed and subsequently perfused to mouse liver via portal vein under anesthesia. After perfusion, the liver was removed carefully and loosened to cell suspension in 50 mL of 0.2 mg/mL protease (Sigma-Aldrich)/0.1 µg/mL DNase I (Sigma-Aldrich) solution in DMEM. The cell suspension was incubated at 37 °C for 20 min while stirring and centrifuged at 50 g for 5 min. The pellet were collected in 0.1 µg/mL DNase I solution in DMEM, washed once and collected in DMEM as hepatocytes fraction. To perform Nycodenz density gradient centrifugation, the cell suspension was layered over 8.2% Nycodenz (Sigma-Aldrich) solution and centrifuged at 1400×*g* and 4 °C for 30 min. Following centrifugation, the intermediate layer over the 8.2% Nycodenz solution was collected in DMEM as HSCs fraction^45^.

### Flow cytometry and cell sorting

After blocking with anti-FcR (CD16/32, BD Biosciences Pharmingen, NJ, USA) for 5 min, the cells were incubated with specific fluorescence-labeled Ab and/or 7-AAD (BD Biosciences Pharmingen)/Fixable viability dye (Thermo Fisher Scientific K.K, Tokyo, Japan) at 4 °C for 20 min, followed by permeabilization and intracellular staining with anti-Foxp3 Ab and anti-Helios Ab in the case of Treg staining. The following primary Abs were used: anti-mouse CD45 (BioLegend, CA, USA, BV510, clone 30-F11), anti-mouse CD45.2 (BD Biosciences Horizon, NJ, USA, FITC/BV510, clone 104), anti-mouse CD45.1 (BD Biosciences Pharmingen/BioLegend, FITC/PE-Cy7, clone A20), anti-mouse TCR-β (BD Biosciences Pharmingen/BioLegend, PerCP-Cy5.5/APC/APC-Cy7, clone H57-597), anti-mouse NK1.1 (BioLegend PE-Cy7, clone PK136), anti-mouse CD4 (BD Biosciences Pharmingen/BD Biosciences Horizon, NJ, USA, FITC/BV510, clone RM4-5), anti-mouse CD8α (BD Biosciences Pharmingen/BD Biosciences Horizon, FITC/PerCP-Cy5.5/APC/APC-Cy7, clone 53-6.7), anti-mouse CD69 (Thermo Fisher Scientific K.K, FITC, clone H1.2F3), anti-mouse CD103 (BioLegend, BV421, clone 2E7), anti-mouse CXCR3 antibody (Thermo Fisher Scientific K.K, APC, clone CXCR3-173), anti-mouse CXCR6 (BioLegend, APC, clone SA051D1), anti-mouse CD62L (BD Biosciences Pharmingen, PE, clone MEL-14), anti-mouse KLRG1 (BioLegend, PerCP-Cy5.5, clone 2F1/KLRG1), anti-mouse CD44 (BioLegend, BV421, clone IM7), anti-mouse Foxp3 (Thermo Fisher Scientific K.K, PE/PerCP-Cy5.5, clone FJK-16s), anti-mouse Helios (BioLegend, APC, clone 22F6), anti-mouse TCRγδ (BioLegend, PerCP-Cy5.5, clone GL3), anti-mouse CD19 (BD Biosciences Pharmingen, PE, clone 1D3), anti-mouse CD11b (BD Biosciences Pharmingen, PE-Cy7/APC-Cy7, clone M1/70), anti-mouse CD11c (BD Biosciences Pharmingen, FITC/PE-Cy7, clone HL3), anti-mouse B220 (BioLegend, PerCP-Cy5.5, clone RA3-6B2), anti-mouse PDCA-1 (BioLegend, APC, clone 129c1), anti-mouse CCR5 (Thermo Fisher Scientific K.K, PE, clone 7A4). Events were acquired with FACS Canto II or Fortessa X-20 (BD, NJ, USA), and analyzed with FlowJo software (Tree Star Inc., OR, USA). Cell sorting was performed using FACS Aria III (BD), and over 95% purity of sorted cells was confirmed. The gating strategy for FACS analysis is presented in Supplementary Fig. 1h, i. Detailed information for the antibodies used in this study is summarized in Supplementary Table 2.

### Serological and histological analysis

Serum alanine aminotransferase (ALT) and total cholesterol (TCHO) levels were measured using DRI-CHEM (Fuji Film, Tokyo, Japan) according to the manufacturer’s instructions. Livers were fixed in 10% formalin and embedded in paraffin. Hematoxylin and eosin (H&E), Masson’s trichrome, and Sirius red staining were carried out using paraffin-embedded liver sections. Samples were observed under the BZ X-700 fluorescence microscope (Keyence). Quantification of Sirius red positive area was performed using BZ-X Analyzer (Keyence, Osaka, Japan).

### Measurement of hydroxyproline concentration

Liver hydroxyproline concentrations were determined by Hydroxyproline Assay Kit (QuickZyme Biosciences, Leiden, Netherlands) following the manufacturer’s recommendation.

### qPCR analysis

Total RNA was extracted from cells using RNeasy Mini kit (QIAGEN, Venlo, Netherlands) or TRIzol reagent (Thermo Fisher Scientific K.K). Complementary DNA was synthesized by reverse transcription using the iScript cDNA Synthesis kit (Bio-Rad, CA, USA). Real-time PCR was then performed with TaqMan Universal Master Mix and the following predesigned primers and probes: *Col1a1* (Mm00801666_g1), *Col1a2* (Mm00483888_m1), *Acta2* (Mm00725412_s1), *Timp1* (Mm01341361_m1), *Desmin* (Mm00802455_m1), *Spp1* (Mm00436767_m1), *Il15* (Mm00434210_m1), *Ccl3* (Mm00441258_m1), *Ccl4* (Mm00443111_m1), *Ccl5* (Mm01302427_m1), *Ccr5* (Mm001216171_m1), and *Fas* (Mm00433237_m1) using StepOne Plus systems (Applied Biosystems, CA, USA). The level of target gene expression was normalized against *Gapdh* expression in each sample. Detailed information for the TaqMan probes is summarized in Supplementary Table 3.

### Single-cell RNA sequencing

CD8^+^ T cells (CD45^+^TCRβ^+^NK1.1^**−**^CD8α^+^) were sorted from the pooled liver mononuclear cells of four mice (10,000 cells each) and loaded into Chromium Controller (10X Genomics,). RNA-seq and TCR-seq libraries were then prepared using Chromium Single Cell 3′ Reagent Kit v2, and 5′ library construction and VDJ enrichment kit v1 according to manufacturer’s instructions (10X Genomics, CA, USA). The generated scRNA-seq libraries were sequenced using a 150 cycle (paired-end reads) with a HiSeqX (Illumina, CA, USA) and DNBSEQ-G400 (MGI Tech, Shenzhen, China).

Sequence reads from all samples were processed by Cell Ranger (10X Genomics). Seurat version 3.1.1^46^ was used to aggregate and analyze the processed data by following the official vignettes (https://satijalab.org/seurat/articles/integration_introduction.html). Specifically, PCA analysis was performed to identify clusters, and 17 gene clusters (0–16) were projected on Uniform Manifold Approximation and Projection (UMAP) space with the normalized gene expression. A gene expression heatmap showing the unbiased generation of the top 10 differentially expressed genes for each cluster (Fig. 2b). For scTCR-seq, every unique clonotype defined using Cell Ranger was identified across all library annotations files and clonotype information was integrated by “add_clonotype” function (https://ucdavis-bioinformatics-training.github.io/2020-Advanced_Single_Cell_RNA_Seq/data_analysis/VDJ_Analysis_fixed). Clone size and frequencies were projected on UMAP (Fig. 4h) and a bar chart (Fig. 4i).

### Parabiosis

To assess the hemodynamics of liver CD8^+^ T cells, we performed parabiosis surgery between sex- and age-matched Ly5.2+ RES (5 weeks from switching to ND) mice and Ly5.1+ RES (5 weeks from switching to ND) mice. Briefly, mice were anesthetized prior to surgery, and incisions were made in the skin on the opposing flanks of the donor and recipient animals. Surgical sutures were used to bring the body walls of the two mice into direct physical contact. The outer skin was then attached with surgical staples^47^. Two weeks after the parabiosis surgery, mice were killed. Peripheral blood CD45^+^ cells and liver CD8^+^ T cells derived from Ly5.1 mice were analyzed using FACS as described above.

### Bulk RNA-seq

Total RNA was isolated from CD8^+^ T cells using TRIzol reagent (Thermo Fisher Scientific K.K) and Direct-zol RNA Microprep (Zymoresearch, CA, USA). RNA-seq was performed by amplifying RNA samples using a SMART-Seq v4 Ultra Low Input RNA kit for Sequencing (Illumina) according to the manufacturer’s instructions. Library preparation was performed using a TruePrep™ DNA Library Prep Kit V2 for Illumina. Libraries were sequenced by Illumina Hiseq X Ten on the 150-bp paired-end mode. The sequenced reads were mapped to the mouse reference genome (NCBI mm10) and read counts were determined by salmon ver. 0.14.1 software pipeline^48^. Normalization of read counts to trimmed mean of M values (TMM) by edgeR and PCA analysis was determined with the TCC-GUI tool (https://github.com/swsoyee/TCC-GUI) and R package. Heat map, k-means clustering, gene ontology (GO) enrichment analysis, and volcano plot were generated using TMM values with the iDEP.91 tool (http://bioinformatics.sdstate.edu/idep/) and R package.

### Antibody/inhibitor treatment during NASH resolution

All groups were normalized after HFHC diet treatment for 24 weeks, or MCD treatment for 7 weeks by body weight and serum ALT. Mice fed the HFHC diet were administered antibodies from 2 weeks to 5 weeks after switching to a ND, while those fed a MCD diet were administered antibodies from days 2–7 after switching to a ND. To deplete CD8^+^ T cells, the anti-mouse CD8α antibody (400 µg/head; Bioxcell NH, USA, clone 2.43) or the isotype control (Bioxcell) was administered intraperitoneally once every 3 days for HFHC-fed mice, or on day 2 and day 5 for MCD-fed mice. To deplete NKT and NK cells, an anti-mouse NK1.1 antibody (200 µg/head; Bioxcell, clone PK136)/anti-mouse CD4 antibody (200 µg/head; Bioxcell, clone GK1.5) or isotype controls (Bioxcell) were administered intraperitoneally once every 3 days. To neutralize IL-15, anti-mouse IL-15 antibody (300 µg/head; Bioxcell, clone AIO.3) or isotype controls (Bioxcell) were administered intraperitoneally once every 3 days. To neutralize FasL, anti-mouse FasL antibody (200 µg/head; Bioxcell, clone MFL3) or isotype controls (Bioxcell) were administered intraperitoneally once every 3 days. To deplete CXCR3^+^ cells, anti-mouse CXCR3 antibody (500 µg/head; Bioxcell, clone CXCR3-173) or isotype controls (Bioxcell) were administered intraperitoneally once every 3 days. Detailed information for the antibodies used in this study is summarized in Supplementary Table 2.

### cDC or pDC depletion in CD11c- or Siglec-H DTR mice during NASH resolution

All groups were normalized after HFHC diet treatment for 24 weeks by body weight and serum ALT. Diphtheria toxin (DT; Sigma Aldrich) was then administered from 2 weeks to 5 weeks after switching to ND. To deplete CD11c^+^ cDCs, DT (100 ng/head in PBS) was administered intraperitoneally twice per week to *Itgax*^dtr/+^ mice and littermate controls. To deplete pDCs, DT (1 µg/head in PBS) was intraperitoneally administered twice per week to *Siglech*^dtr/+^ mice and littermate controls.

### CD8^+^ T cell adoptive transfer to MCD diet mice

To induce NASH, a MCD diet was fed to Ly5.2 mice for 8 weeks. Liver CD8T cells or non-CD8T cells were isolated from Ly5.1 HFHC or RES (5 weeks) mice using anti-CD8α microbeads (Miltenyii Biotec, Japan K.K), and the isolated cells (2 × 10^6^ cells) were intravenously transferred to MCD diet treated mice on days 1, 6, and 11 starting from week 6 for 2 weeks. To isolate liver CD8^+^ Trm cells, CD45^+^TCRβ^+^NK1.1^**−**^CD8α^+^ CD44^+^CD62L^**−**^ KLRG1^**−**^CD69^+^ cells were sorted by FACS Aria III (BD). To avoid Trm cell apoptosis caused by isolating and transferring procedures, anti-ARTC2 nanobody (50 µg/mouse; BioLegend, clone 16 + s) was inoculated to donor mice 30 min before killing^49^.

### Migration assay

Migration assays were performed using 8 μm pore 96-well Transwell plates (Corning, NY, USA). HSCs and liver CD8^+^ T cells were separated from RES or HFHC (5 weeks after switching to ND or keeping the HFHC diet) mice, and HSCs were cultured on the upper chamber of the transwell (1 × 10^4^ /well). Following FACS sorting, each CD8^+^ T cell subset (1 × 10^5^/200 µL/well) was incubated for 24 h with CD3/28 stimulation (Thermo Fisher Scientific K.K), and the supernatant was applied to the lower chamber with or without CCR5 inhibitor (Marovenic; MVC, Selleckchem, TZ, USA). After 24 h, the cell numbers in the lower chamber were counted.

### Cytotoxic assay of CD8^+^ Trm cells

HSCs or hepatocytes were separated from RES or HFHC (5 weeks after switching to ND or keeping HFHC treatment) mice and seeded into 96-well plates (2 × 10^4^ cells/well). FACS-sorted liver CD8^+^ T naïve cells or CD8^+^ Trm cells (0.5, 1, 2, and 4 × 10^4^ cells/well) from RES or HFHC (5 weeks after switching to ND or keeping the HFHC treatment) mice were co-cultured with HSCs or hepatocytes for 8 h at 37 °C. Culture supernatants were then retrieved and LDH-based cytotoxicity was determined by Cytotox 96 Non-radioactive cytotoxicity assay (Promega, WI, USA) according to the manufacturer’s instructions. To assess the effect of each inhibitor on CD8^+^ T cell cytotoxicity, 1 × 10^4^ CD8^+^ Trm cells and HSCs derived from RES (5 week after switching to ND) mice were co-cultured with anti-FasL (1 µg/mL; BioLegend, clone MFL4, cat 106707), anti-IFN-γ Ab (1 µg/mL; BD Biosciences Pharmingen, clone XMG1.2, cat 559065), anti-PD-1 Ab (1 µg/mL; BioLegend, clone 29 F.1A12, cat 135246), anti-TIGIT Ab (1 µg/mL; BioLegend, clone A17200C, cat 622203), Concanamycin A (Perforin/Granzyme inhibitor; Sigma Aldrich, 100 nM), Granzyme B inhibitor II (Merck Millipore, MA, USA, 100 nM), control IgG (1 µg/mL), or vehicle control (DMSO) for 8 h at 37 °C. Culture supernatants were then retrieved and cytotoxicity was determined as described above.

### Preparation of vitamin A-coupled liposomes carrying Ccr5-siRNA

*Ccr5* or control siRNA bearing vitamin A-coupled liposomes were prepared using *Ccr5* or control siRNA (Nippon Gene, Tokyo, Japan) and liposomes containing cationic lipids (LipoTrustTM SR, Hokkaido System Science, Sapporo, Japan) mixed with vitamin A (Sigma Aldrich). In brief, liposomes containing cationic lipids (LipoTrust SR; Hokkaido System Science, Sapporo, Japan) were prepared at a concentration of 1 mM (cationic lipids) using RNase-free double-distilled water, according to the manufacturer’s protocol. To prepare vitamin A-coupled liposomes, vitamin A (200 nmol) (Sigma, St. Louis, MO, USA) and liposomes (100 nmol as cationic lipids) were mixed by vortexing at room temperature. The Ccr5-siRNA and control-siRNA (Nippon Gene, Tokyo, Japan) were prepared at a concentration of 580 pmol/µL, and mixed well with vitamin A-coupled liposomes. After incubation at 37 °C for 5 min, free vitamin A was removed from the liposome preparations using a micro-partitioning system (Vivaspin 2 concentrator 30,000 MWCO PES; Vivascience, Göttingen, Germany)^50^.

### Immunohistochemistry

Antigens were activated by autoclaving and blocked using Block Ace (Megmilk Snow Brand Co., Tokyo, Japan) followed by primary antibody reaction at room temperature for 4 h. A mouse anti-mouse CD8 antibody (1/50, Santa Cruz, CA, USA, sc-70802), rabbit anti-mouse Desmin antibody (1/100, Abcam, Cambridge, England, ab15200), mouse anti-mouse Desmin antibody (1/100, Abcam, ab6322), anti-mouse cleaved caspase 3 antibody (1/100, R& D systems, MN, USA, MAB835), anti-mouse FasL antibody (1/100, Sino Biological, Beijing, China, 101984), anti-mouse Fas antibody (1/100, R& D systems, AF435), anti-mouse Granzyme B antibody (1/100, R& D systems, AF1865), and anti-mouse Perforin antibody (1/100, Abcam, Ab16074) were used as primary antibody. After washing with PBS, the sections were incubated with Alexa Fluor 488–, Alexa Fluor 555–, or Alexa Fluor 647–labeled secondary antibodies (Abcam) at room temperature for 2 h. In situ apoptosis detection kit (Takara Bio, Shiga, Japan) was used for TUNEL staining. Tissue samples were observed under the fluorescence microscope BZ X-700 (Keyence) or the LSM 710 confocal laser scanning microscope (Carl Zeiss, Gena, Germany). HSCs were counted on randomly selected 5 fields of 540 µm × 720 µm at ×40 magnification and normalized to 1 field count for each liver section using BZ X-700. TUNEL + HSCs, CD8^+^ T cells, and CD8^+^ T cells co-localized with HSCs were counted on randomly selected 5 fields of 100 µm × 100 µm at ×200 magnification and normalized to 1 field count for each liver section using LSM 710. CD8^+^ T cells attached to HSCs were defined as CD8^+^ T cells co-localized with HSCs. Quantification of Fas^+^ HSCs positive area was performed using Zen software (Carl Zeiss) and ImageJ (NIH). Detailed information for the antibodies used in this study is summarized in Supplementary Table 2.

### TCR Vβ repertoire analysis

The TCR Vβ repertoire of liver CD8^+^ Trm cells was determined by FACS analysis using anti-mouse TCR Vβ Screening panel (BD Biosciences Pharmingen) according to the manufacturer’s instructions.

### Patients

Liver tissue samples were obtained from patients with NASH (*n* = 13) and patients affected by hepatic metastasis of gastrointestinal cancer with normal liver function (*n* = 5) as the control group. Clinical characteristics and baseline demographics are presented in Supplementary Table S1. Written informed consent was obtained from all participants.

### Immunohistochemistry of human liver tissues

IHC was performed using a Bond-Max automated immunohistochemical staining machine (Leica Microsystems, Milton Keynes, UK). Evaluation of CD69^+^ CD8^+^ T cells was performed using double fluorescent immunohistochemistry. Anti-human CD8 monoclonal Ab (Nichirei, Japan, clone C8/144B) and anti-human CD69 monoclonal antibody (Abcam, clone EPR21814) were used as primary Abs. The slides were scanned to obtain multi-colored whole-slide images using a NanoZoomer 2.0HT scanner (Hamamatsu Photonics K.K., Shizuoka, Japan) with a ×40 objective lens, according to the manufacturer’s instructions. The slides were further stained with H&E and rescanned to obtain merged images of H&E images on top of the pre-scanned multi-colored images^51^. To quantify CD69^+^ CD8^+^ T cells, all portal areas were checked for merged CD69^+^ CD8^+^ T cells using NDP.view2 software (Hamamatsu Photonics K.K), and the highest number in 1-mm^2^ areas was determined as the maximum cell count. Detailed information for the antibodies used in this study is summarized in Supplementary Table 2.

### Study approval

Animal Ethics Committee of Keio University approved all animal studies. The institutional review board of Keio University School of Medicine approved all human studies (No. 20120395 and No. 20040034) according to the guidelines of the 1975 Declaration of Helsinki (2008 revision). The human study participants were prospectively recruited, and each participant provided prior written informed consent for blood sampling, study participation, and analysis of clinical data. All human study participants received standard care and treatment according to their clinical presentations.

### Statistics and reproducibility

Statistical analyses were performed using GraphPad Prism software version 7.0 g and 8.0 g (GraphPad software Inc. CA, USA). Differences between two groups were evaluated using two-sided unpaired Student’s *t* tests. Comparisons of more than two groups was performed with one-way ANOVA followed by Tukey-Kramer’s multiple comparison test. For all analyses, significance was accepted at a 95% confidence level (*p* < 0.05). Each experiment was replicated more than two times with reproducible results.

### Reporting summary

Further information on research design is available in the Nature Research Reporting Summary linked to this article.

## Supplementary information

Supplementary information

Reporting Summary

## Data Availability

The raw scRNA sequencing data have been deposited in the NCBI GEO database under accession number GSE176210. The raw bulk-RNA sequencing data have been deposited in the DNA Data Bank of Japan under accession number DRA012190. Source data are provided with this paper as a Source Data file. All other data are available from the corresponding authors upon reasonable request. Source data are provided with this paper.

## Source data

Source data file
