# Supplementary material for: CD8+ tissue-resident memory T cells promote liver fibrosis resolution by inducing apoptosis of hepatic stellate cells
Source: Nat Commun. 2021 Jul 22;12:4474. doi: 10.1038/s41467-021-24734-0 (PMC8298513; doi:10.1038/s41467-021-24734-0)
Supplement: Supplementary file 1 — Supplementary information [file 41467_2021_24734_MOESM1_ESM.pdf]

**Supplementary Information for**

# CD8+ tissue-resident memory T cells promote liver fibrosis resolution by inducing apoptosis of hepatic stellate cells

Yuzo Koda,[1,2] Toshiaki Teratani,[1] Po-Sung Chu,[1] Yuya Hagihara,[1] Yohei Mikami,[1] Yosuke Harada,[1] Hanako Tsujikawa,[3] Kentaro Miyamoto,[1] Takahiro Suzuki,[1] Nobuhito Taniki,[1] Tomohisa Sujino,[1] Michiie Sakamoto,[3] Takanori Kanai,[1,4*] Nobuhiro Nakamoto[1*]

[1]Division of Gastroenterology and Hepatology, Department of Internal Medicine, Keio University School of Medicine, Tokyo, Japan
[2]Mitsubishi Tanabe Pharma Corporation, Kanagawa, Japan
[3]Department of Pathology, Keio University School of Medicine, Tokyo, Japan
[4]Japan Agency for Medical Research and Development, AMED, Tokyo, Japan

*Address correspondence to Nobuhiro Nakamoto (nobuhiro@z2.keio.jp) and Takanori Kanai (takagast@z2.keio.jp), Division of Gastroenterology and Hepatology, Department of Internal Medicine, Keio University School of Medicine, Tokyo 1608582, Japan.

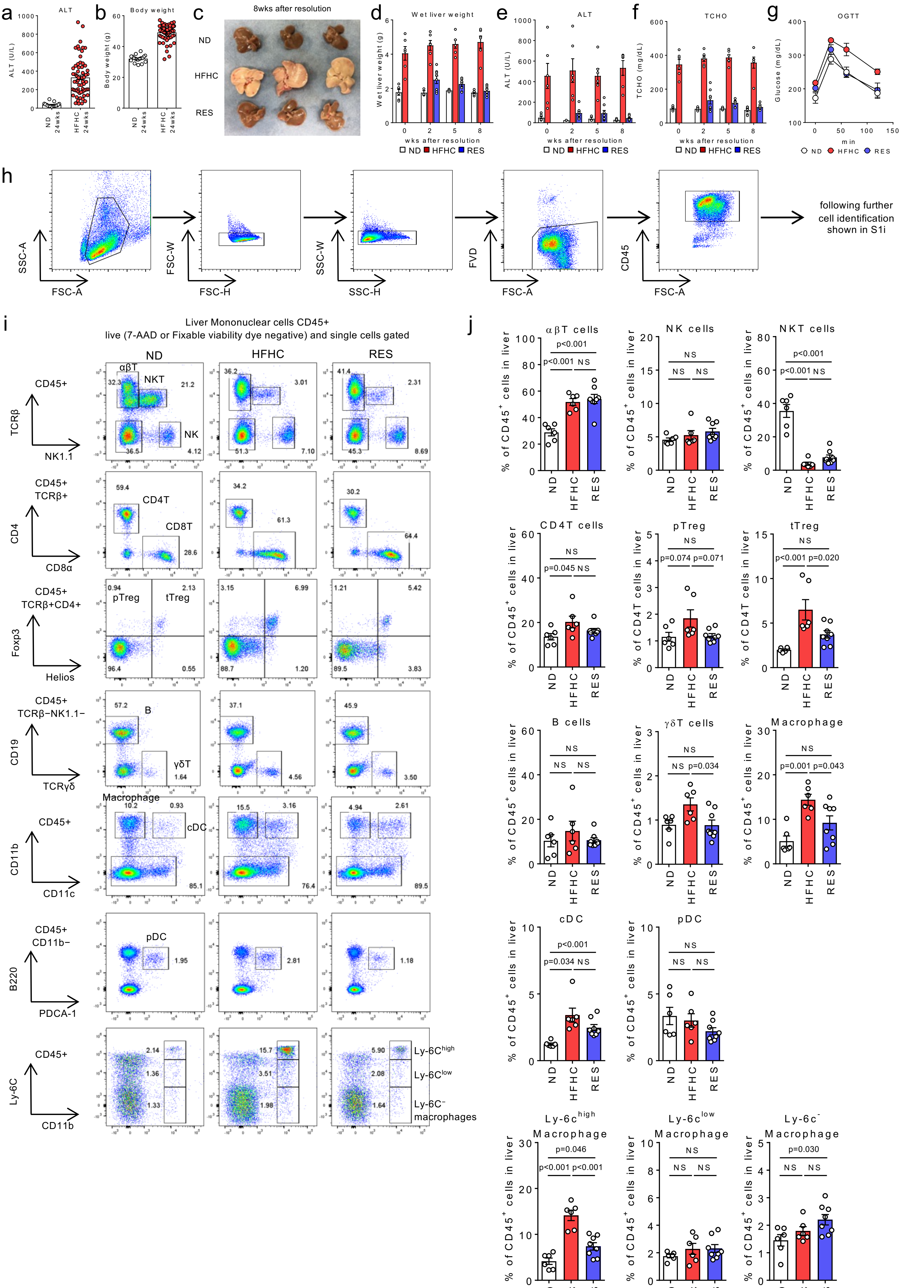


Figure S1 Koda et. al.

**Supplementary Figure 1. Compensated data of HFHC-induced NASH and liver fibrosis resolution models.** **a**, Serum ALT levels and **b**, body weight of HFHC-induced NASH mice (n=17 mice for ND 24 weeks group and n=63 mice for HFHC 24 weeks group). **c**, Representative gross photographs of livers. **d**, Wet liver weight, **e**, Serum ALT levels, **f**, Serum TCHO, and **g**, OGTT of ND, HFHC, and RES mice (**d-f**; n=3-6 mice for ND group, n=6 mice for HFHC group, and n=8 mice for RES group. **g**; n=4 mice for ND and RES groups, and n=3 mice for HFHC group). **h** and **i**, Gating strategy for FACS analysis. **j**, Frequency of the indicated immune cells in $CD45^{+}$ liver MNCs (n= 6 mice for ND and HFHC groups, and n=8 mice for RES group). Data are presented as mean ± SEM. One-way ANOVA with Tukey's multiple comparisons post-hoc test was applied.

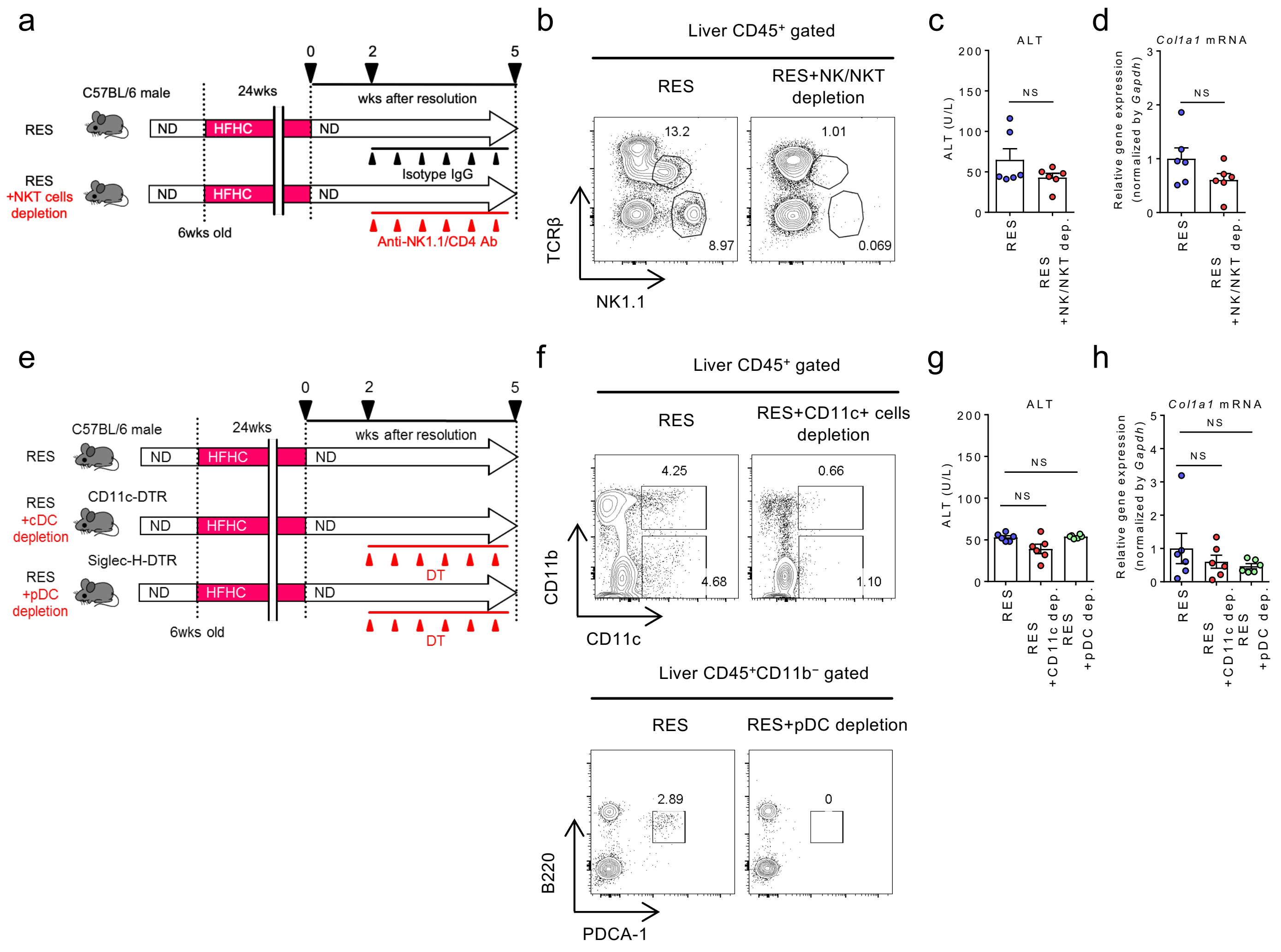


**Supplementary Figure 2. CD11c⁺ myeloid cells, pDCs, NK cells, and NKT cells play no direct roles in NASH resolution.** **a**, Study design: resolution induced mice were intraperitoneally treated with isotype control (RES group) or anti-NK1.1 antibody and anti-CD4 antibody (RES+NK/NKT depletion group) once every three days for three weeks starting at week 2 following diet switch (n=6 mice per group). **b**, Representative TCRβ/NK1.1 staining of $CD45^+$ gated liver MNCs. **c**, Serum ALT levels. **d**, *Col1a1* mRNA levels. **e**, Study design: resolution induced mice (wild-type (WT), CD11c-DTR, or Siglec-H-DTR background) were either untreated or intraperitoneally treated with diphtheria toxin (DT) twice per week for three weeks starting at week 2 following diet switch (n = 6 micec per group). **f**, Representative TCRβ/NK1.1 staining of $CD45^+$ gated liver MNCs (upper) and B220/PDCA-1 staining of $CD45^+CD11b^-$ gated liver MNCs (lower). **g**, Serum ALT levels. **h**, *Col1a1* mRNA levels. Data are presented as mean ± SEM. Two-sided unpaired Student's *t*-test (**c** and **d**) or one-way ANOVA with Tukey's multiple comparisons post-hoc test (**g** and **h**) was applied.

Figure S2 Koda et. al.

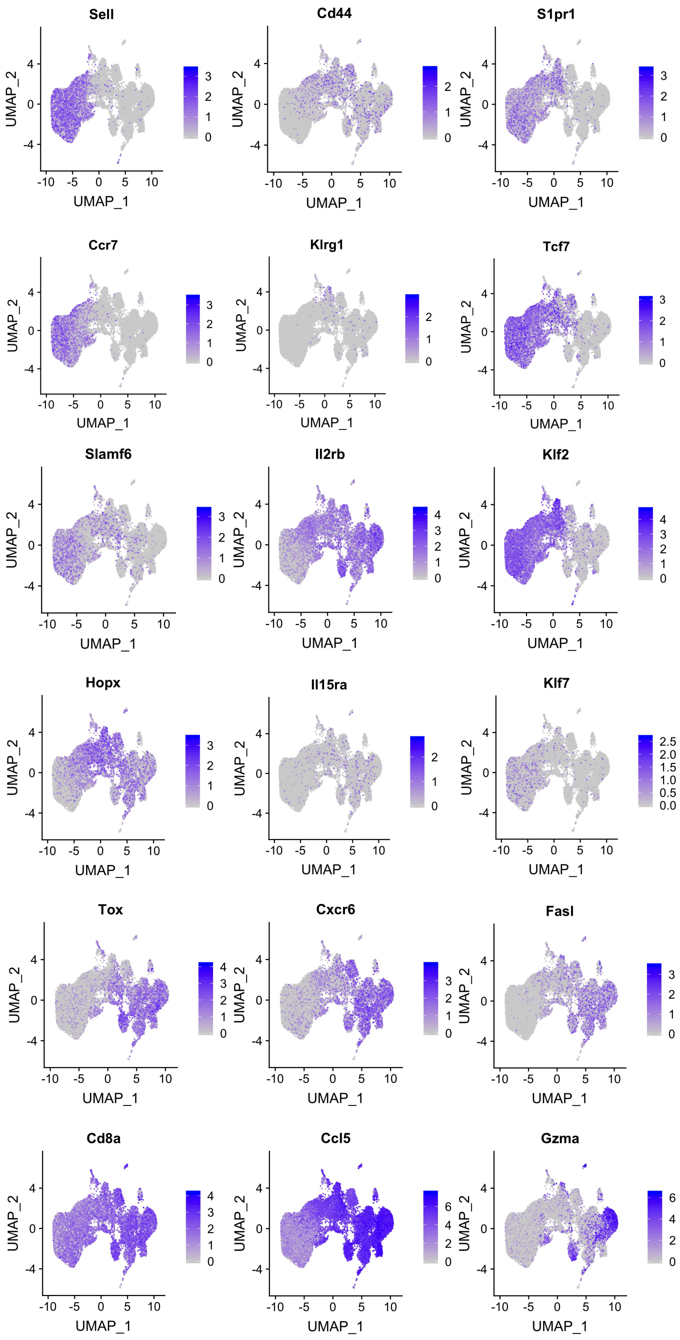


**Supplementary Figure 3. Compensated data for scRNA-seq analysis of liver CD8⁺ T cells.** Expression levels of the specified marker genes on the UMAP plots of combined liver CD8⁺ T cells.

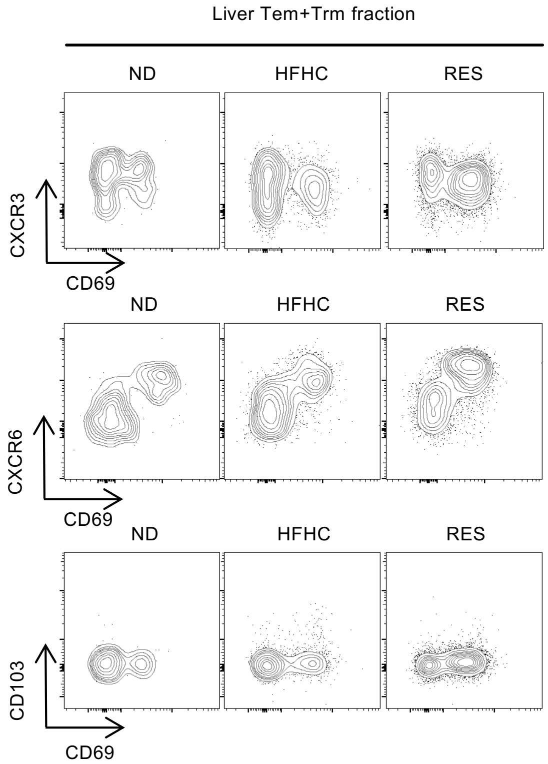


**Supplementary Figure 4. Compensated data for FACS analysis of liver CD8⁺ T cells.** Representative CXCR3/CD69 (upper), CXCR6/CD69 staining (middle), and CD103/CD69 staining (lower) of $CD45^{+}TCR\beta^{+}NK1.1^{-}CD8\alpha^{+}CD44^{+}CD62L^{-}$ gated (Tem+Trm) liver MNCs derived from ND, HFHC, and RES (5 weeks) mice.

Figure S4 Koda et. al.

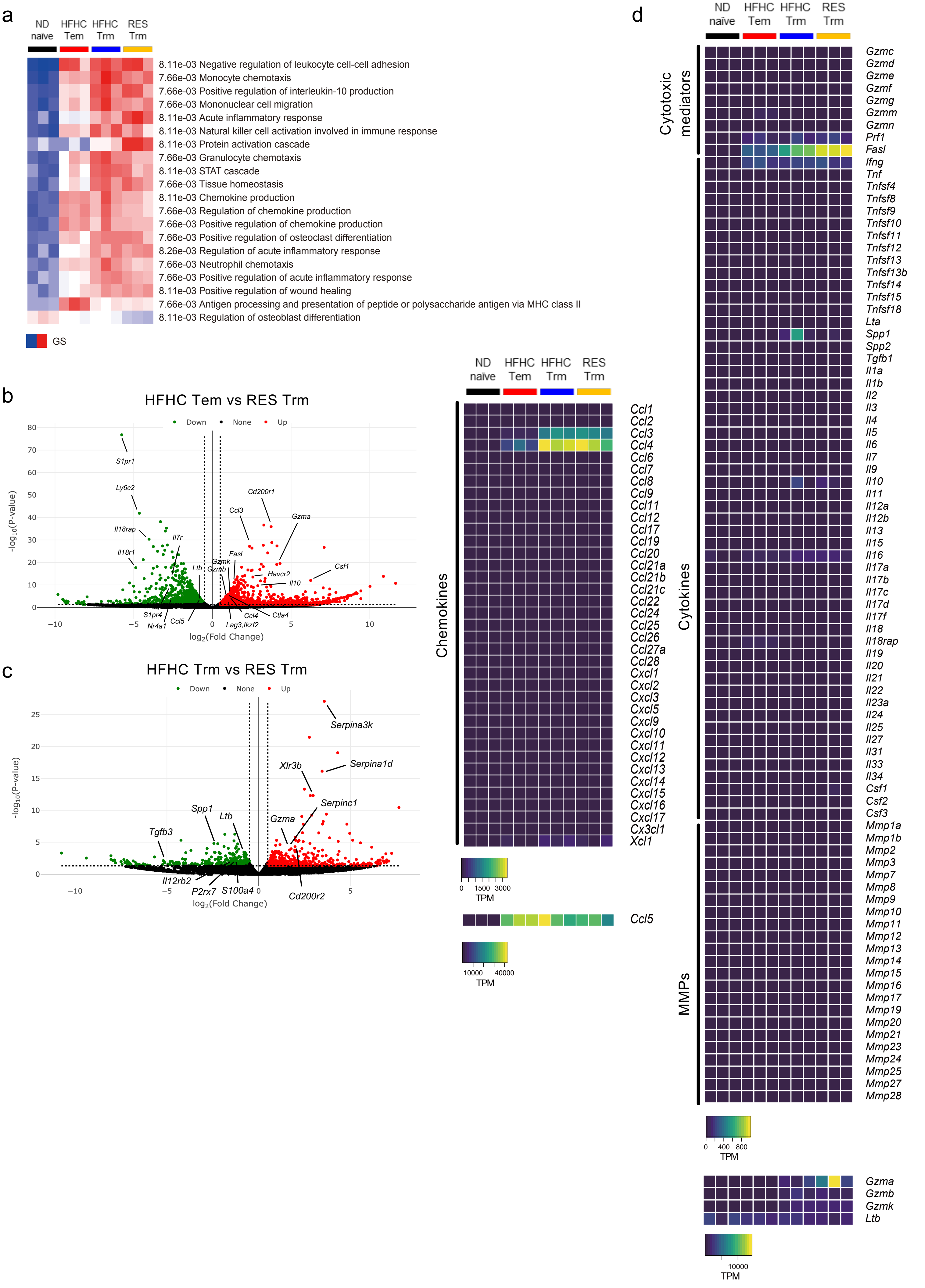


Figure S5 Koda et. al.

**Supplementary Figure 5. Compensated data for bulk RNA-seq analysis of liver CD8$^+$ T cells. a,** Heat map from Gene ontology (GO) enrich analysis between the indicated groups. GO terms enriched in top 2000 significantly different genes were shown (top 20, FDR<0.1). **(b and c)** Volcano plot of HFHC Tem vs RES Trm (**b**), and of HFHC Trm vs RES Trm (**c**). Red and green dots mean significantly higher and lower genes in RES Trm (P<0.01, Fold change> $\log_2 1.5$), respectively. **d,** Heat map showing the absolute transcript levels of genes related to chemotaxis (left) and inflammatory pathways (right). Source data are provided as a Source data file.

a

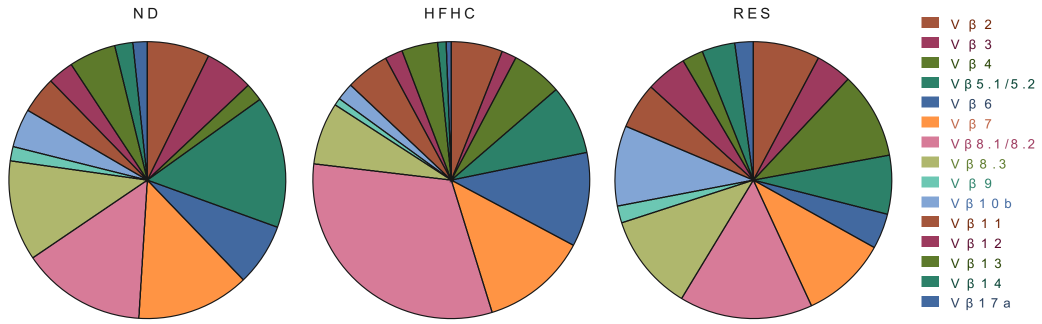


b

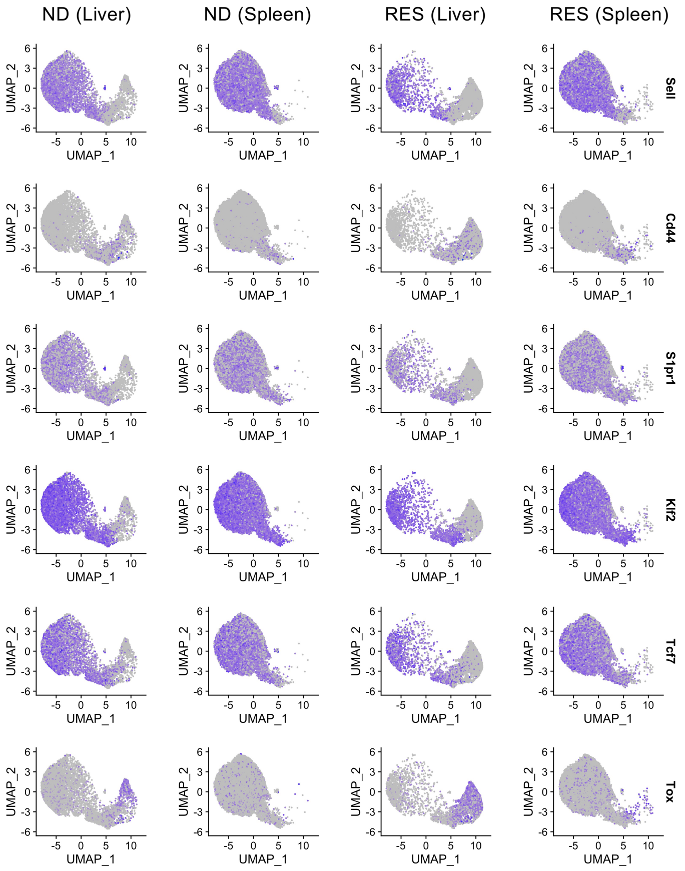


c

| (%) | ND (Liver) | ND (Spleen) | RES (Liver) | RES (Spleen) |
|---|---|---|---|---|
| **clonotype1** | 4.81 | 0.08 | 39.94 | 0.26 |
| **clonotype2** | 0.98 | 0.05 | 9.06 | 0.19 |
| **clonotype4** | 0.55 | 0.04 | 3.46 | 0.10 |
| **clonotype3** | 0.45 | 0.04 | 3.40 | 0.16 |
| **clonotype5** | 0.61 | 0.03 | 2.89 | 0.07 |
| **clonotype6** | 0.42 | 0.04 | 1.74 | 0.07 |
| **clonotype7** | 0.54 | 0.04 | 1.35 | 0.05 |
| **clonotype8** | 0.33 | 0.03 | 1.31 | 0.06 |
| **clonotype9** | 0.30 | 0.02 | 1.08 | 0.06 |
| **clonotype10** | 0.21 | 0.02 | 1.00 | 0.04 |
| **Others** | 90.82 | 99.62 | 34.76 | 98.95 |

**Supplementary Figure 6. TCR repertoire analysis of liver CD8⁺ Trm cells and compensated data for scTCR RNA-seq analysis of liver and spleen CD8⁺ T cells. a**, Frequency of 15 different Vβ families in CD8⁺ Trm cells of ND, HFHC, and RES (5weeks) mice (n=6 mice for ND and HFHC groups; n=8 mice for RES group) using FACS analysis. **b,** Expression levels of the specified marker genes on the UMAP plots in each cell subset. **c,** Percent values of each of the top 10 and other TCR clonotypes. Source data are provided as a Source data file.

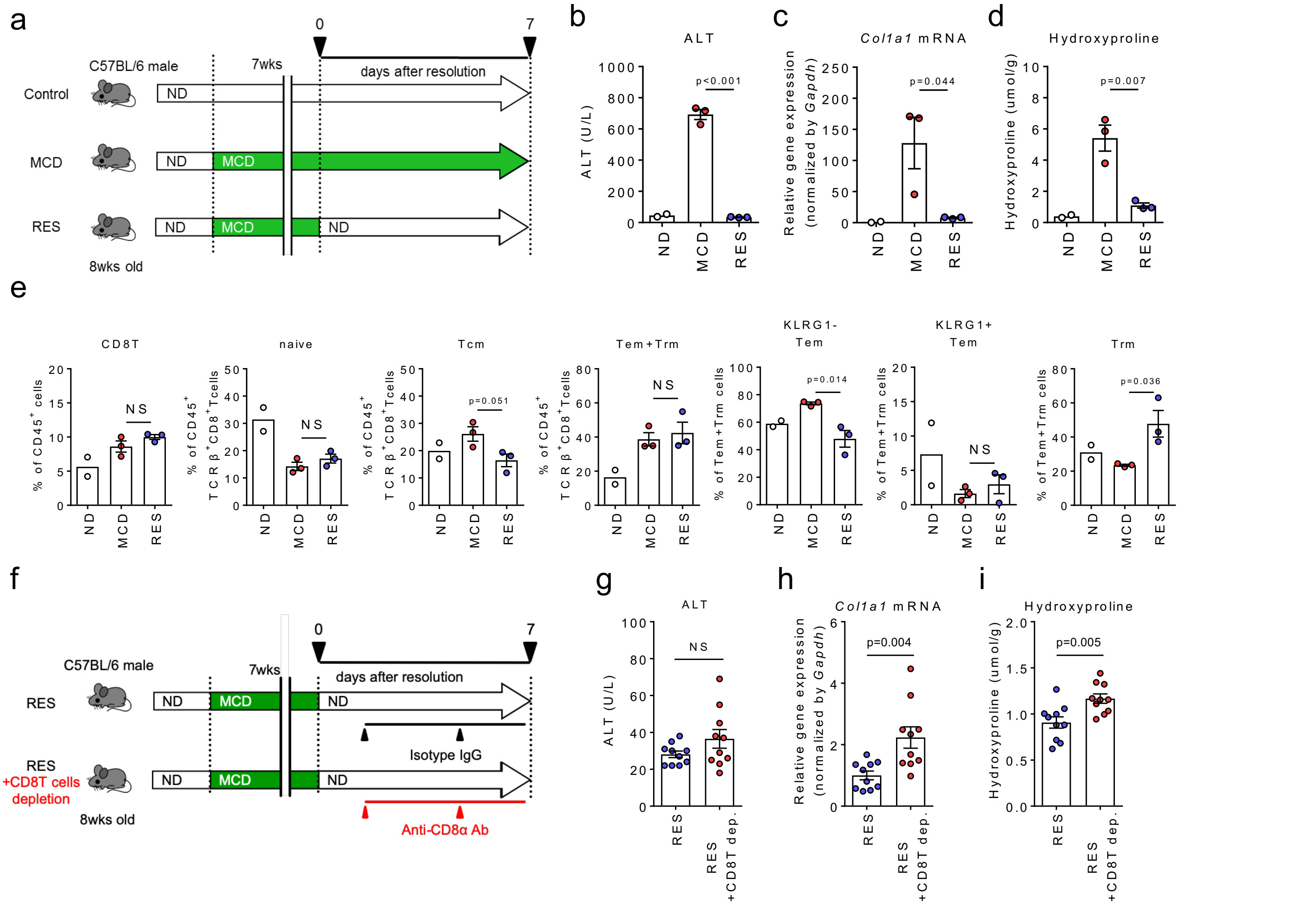


**Supplementary Figure 7. CD8$^+$ T cells play a direct role in MCD-induced liver fibrosis resolution. a**, Study design: Male C57BL/6 mice were fed a ND or MCD diet for seven weeks, after which ND-treated mice were continuously fed a ND (ND group) for one week. MCD diet-treated mice were fed MCD diet (MCD group) or switched to ND (resolution; RES group) for one week to induce resolution (n=2 mice for ND group, and n=3 mice for MCD and RES groups). **b**, Serum ALT levels. **c**, *Col1a1* mRNA levels. **d**, Hydroxyproline levels. **e**, Proportion of each CD8$^+$ T cell subset in CD45$^+$ liver MNCs of the indicated groups. **f**, Study design: resolution induced mice were intraperitoneally treated with isotype control (RES group) or anti-CD8α antibody (RES+CD8T depletion group) on day 1 and day 4 after the diet switch (n=10 mice per group). **g**, Serum ALT levels. **h**, *Col1a1* mRNA levels. **i**, Hydroxyproline levels. Data represent mean ± SEM. Two-sided unpaired Student's *t*-test was applied (MCD vs. RES for **b-e**, and RES vs. RES+CD8T dep. for **g-i**).

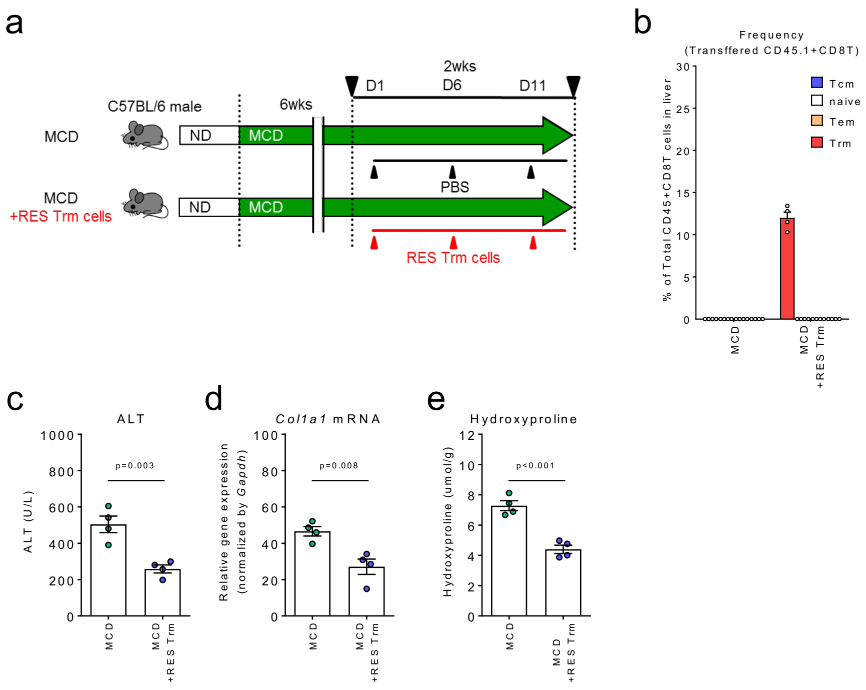


**Supplementary Figure 8. Transfer of sorted CD8⁺ Trm cells ameliorates MCD-induced liver fibrosis. a**, Study design: male C57BL/6 (Ly5.2) mice were fed a MCD diet for eight weeks and were intravenously injected with either PBS or liver CD8⁺ Trm (CD45⁺TCRβ⁺NK1.1⁻CD8α⁺CD44⁺CD62L⁻KLRG1⁻CD69⁺) cells isolated from Ly5.1RES (5 weeks) mice ($2 \times 10^6$ cells each) on days 1, 6, and 11 starting at week six (n=4 mice per group). **b**, Frequency of the transferred CD8⁺ T cell subset in CD45⁺ liver MNCs of the indicated mice groups. **c**, Serum ALT levels. **d**, *Col1a1* mRNA levels. **e**, Hydroxyproline levels. Data are presented as mean ± SEM. Two-sided unpaired Student's *t*-test was applied.

Figure S8 Koda et. al.

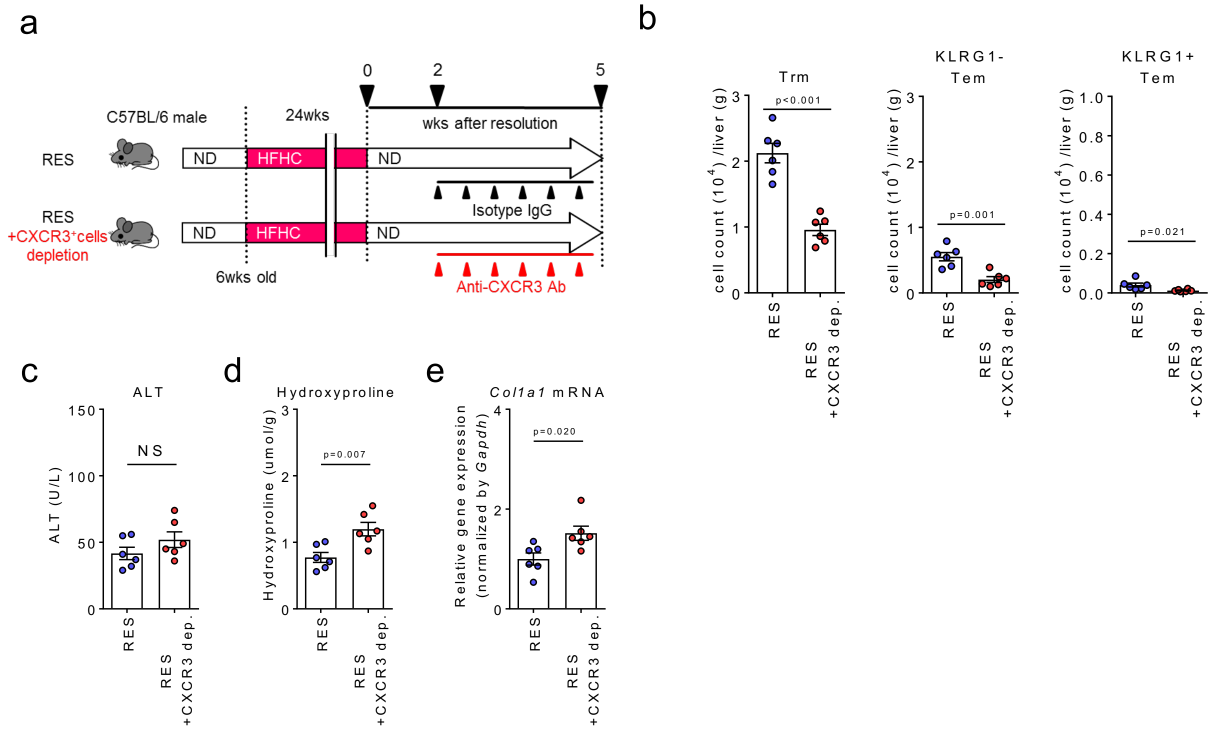


**Supplementary Figure 9. Depletion of CXCR3⁺ cells prevents HFHC-induced liver fibrosis resolution. a**, Study design: RES mice were treated with either anti-CXCR3 antibody (RES+CXCR3⁺ cells depletion group) or the isotype control (RES group) once every three days for three weeks starting at week 2 following diet switch (n=6 mice per group). **b**, Number of CD8⁺ Trm, KLRG1⁻ CD8⁺ Tem, and KLRG1⁺CD8⁺ Tem cells in CD45⁺ liver MNCs. **c**, Serum ALT levels. **d** Hydroxyproline levels. **e**, *Col1a1* mRNA levels. Data are presented as mean ± SEM. Two-sided unpaired Student's *t*-test was applied.

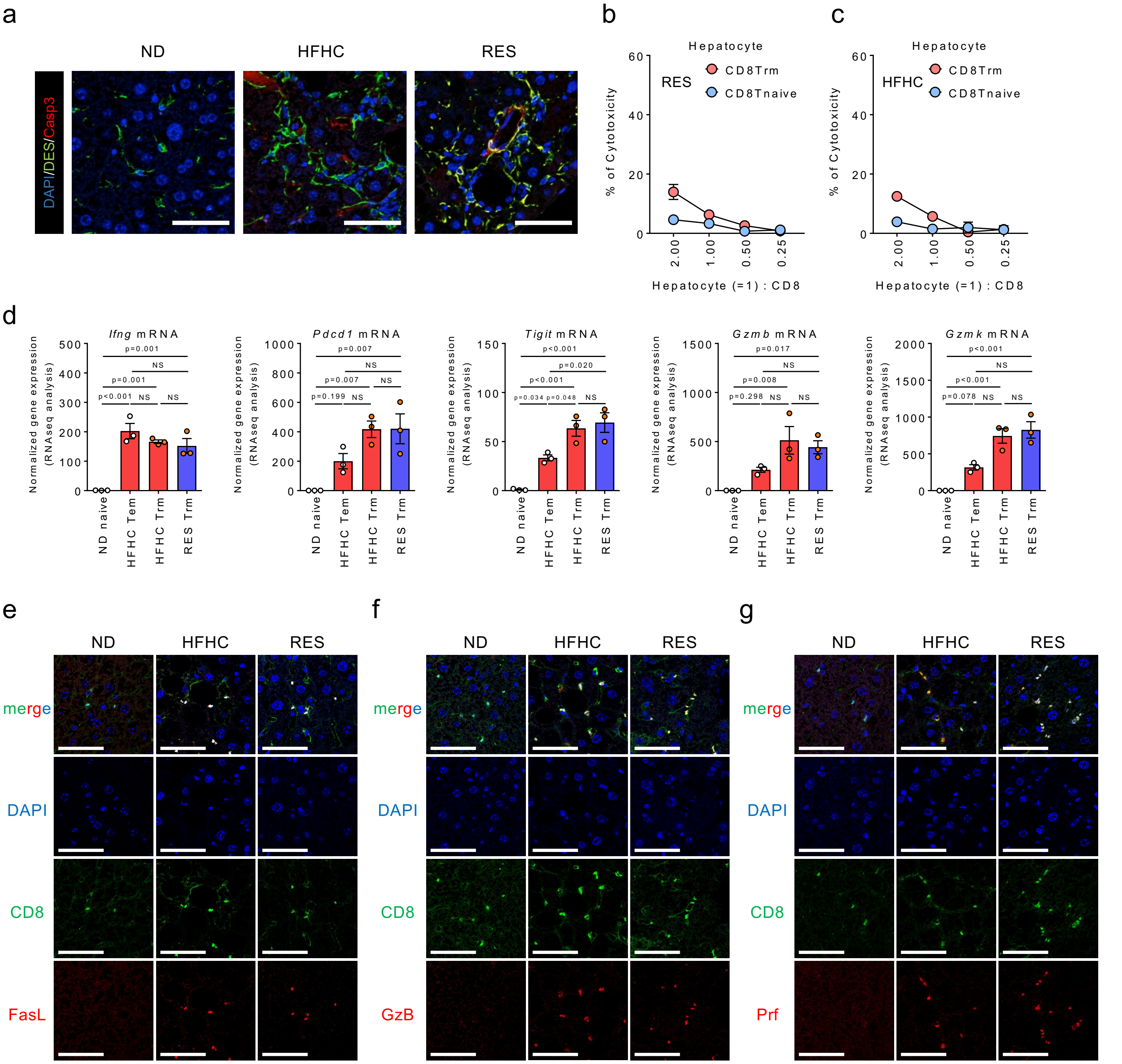


**Supplementary Figure 10. Cytotoxicity of HSCs by CD8⁺ T cells. a,** Representative photomicrographs of liver sections stained with DAPI (blue), anti-desmin (green), and anti-cleaved caspase 3 Ab (red). Scale bars: 50 μm. **b, c,** % cytotoxicity of hepatocytes by CD8 Trm (pink) or CD8 Tnaive (blue) isolated from RES 5weeks mice (**b**) or HFHC mice (**c**) (n=3 biologically independent samples per group). **d,** *Ifng, Pdcd1, Tigit, Gzmb, and Gzmk* mRNA levels in ND naïve, HFHC Tem, HFHC Trm, and RES Trm cells (n=4 mice per group). Data are presented as mean ± SEM. One-way ANOVA with Tukey's multiple comparisons post-hoc test was applied. **e,** Representative fluorescent photomicrographs of liver sections stained with DAPI (blue), anti-CD8 Ab (green), and anti-FasL (red). Scale bars: 50 μm. **f,** Representative fluorescent photomicrographs of liver sections stained with DAPI (blue), anti-CD8 Ab (green), and anti-Granzyme B (Gzb) (red). Scale bars: 50 μm. **g,** Representative fluorescent photomicrographs of liver sections stained with DAPI (blue), anti-CD8 Ab (green), and anti-Perforin (Prf) (red). Scale bars: 50 μm. Photomicrograph data were representative from 4 independent samples and similar results were confirmed (**a, e-g**). Source data are provided as a Source data file.

Supplementary table 1. Clinical characteristics of participants

| Background Characteristics | All participants | Normal | F1-2 | F3-4 |
|---|---|---|---|---|
| N | 18 | 5 | 4 | 9 |
| Sex, F: M | 6:12 | 1:4 | 0:4 | 5:4 |
| Age, yrs | 70.5 [22-87] | 48 [22-72] | 72.5 [66-87] | 75 [58-86] |
| AST, IU/L | 36.5 [16-63] | 20 [16-25] | 43 [36-63] | 43 [26-62] |
| ALT, IU/L | 26.5 [10-84] | 18 [10-25] | 45 [20-84] | 28 [14-64] |
| γ-GTP, IU/L | 51.5 [14-163] | 29 [14-127] | 56.5 [45-84] | 51 [21-163] |
| Platelet count, $x10^4/\mu L$ | 16.8 [6.3-22.6] | 19.1 [18-22.6] | 19.3 [16.6-22.4] | 11.7 [6.3-19] |
| Total bilirubin, mg/dL | 0.7 [0.4-2.4] | 0.6 [0.4-1.3] | 0.7 [0.6-1.0] | 0.8 [0.4-2.4] |
| Serum albumin, g/dL | 4.1 [3.0-4.6] | 4.4 [4.3-4.6] | 4.2 [4.1-4.2] | 3.8 [3.0-4.2] |
| APRI | 0.62 [0.19-2.34] | 0.25 [0.19-0.34] | 0.57 [0.53-0.70] | 0.83 [0.51-2.34] |
| FIB-4 index | 3.1 [0.54-11.0] | 1.0 [0.54-1.94] | 2.47 [2.04-4.12] | 4.45 [2.37-11.0] |

Data show median with ranges.

Table S1 Koda et. al.

Supplementary table 2. List of antibodies used in this study

| | antibody | Clonality | clone number | conjugate | Dilution | vendor | catalog number | Use |
|---|---|---|---|---|---|---|---|---|
| CD8 | anti- mouse CD8 antibody | monoclonal | 6A242 | | 1/50 | Santa Cruz Biotechnology | sc-70802 | IHC |
| Desmin | anti- mouse desmin antibody | monoclonal | | | 1/100 | Abcam | ab15200 | IHC |
| Desmin | anti- mouse desmin antibody | monoclonal | DE-U-10 | | 1/100 | Abcam | ab6322 | IHC |
| cleaved caspase3 | anti- mousecleaved caspase Antibody | monoclonal | 269518 | | 1/100 | R&D | MAB835 | IHC |
| FasL | anti- mouse FasL antibody | polyclonal | | | 1/100 | sinobiological | 101984 | IHC |
| Fas | anti- mouse Fas antibody | polyclonal | | | 1/100 | R&D | AF435 | IHC |
| Granzyme B | anti- mouse Granzyme B antibody | polyclonal | | | 1/100 | R&D | AF1865 | IHC |
| Perforin 1 | anti- mouse Perforin antibody | monoclonal | CB5.4 | | 1/100 | Abcam | ab16074 | IHC |
| CD8 | anti- human CD8 antibody | monoclonal | C8/144B | | 1/100 | Nichirei | 413211 | IHC |
| CD69 | anti- human CD69 antibody | monoclonal | EPR21814 | | 1/100 | Abcam | ab233396 | IHC |
| | anti- rabbit IgG antibody (Secondary antibody) | polyclonal | | Alexa Fluor 488 | | Abcam | ab150077 | IHC |
| | anti- mouse IgG antibody (Secondary antibody) | polyclonal | | Alexa Fluor 555 | | Abcam | ab150062 | IHC |
| | anti- guinea pig IgG antibody (Secondary antibody) | polyclonal | | Alexa Fluor 647 | | Abcam | ab150187 | IHC |
| CD8α | anti- CD8α antibody | monoclonal | 2.43 | | | Bioxcell | BE0061 | in vivo depletion |
| NK1.1 | anti- NK1.1 antibody | monoclonal | PK136 | | | Bioxcell | BE0036 | in vivo depletion |
| CD4 | anti- CD4 antibody | monoclonal | GK1.5 | | | Bioxcell | BP0003-1 | in vivo depletion |
| IL-15 | anti- IL-15 antibody | monoclonal | AIO.3 | | | Bioxcell | BE0315 | in vivo neutralization |
| FasL | anti- FasL antibody | monoclonal | MFL3 | | | Bioxcell | BE0319 | in vivo neutralization |
| CXCR3 | anti- CXCR3 antibody | monoclonal | CXCR3-173 | | | Bioxcell | BE0249 | in vivo depletion |
| FasL | anti1-FasL antibody | monoclonal | MFL4 | | | BioLegend | 106707 | in vitro neutralization |
| IFN-γ | anti- IFN-γ antibody | monoclonal | XMG1.2 | | | BD Biosciences | 559065 | in vitro neutralization |
| PD-1 | anti- PD-1 antibody | monoclonal | 29F.1A12 | | | BioLegend | 135246 | in vitro neutralization |
| TIGIT | anti- TIGIT antiboby | monoclonal | A17200C | | | BioLegend | 622203 | in vitro neutralization |
| CD45.2 | anti- CD45.2 antibody | monoclonal | 104 | FITC | 1/200 | BD Biosciences | 553772 | FACS |
| CD45.2 | anti- CD45.2 antibody | monoclonal | 104 | BV510 | 1/200 | BD Biosciences | 109838 | FACS |
| CD45.1 | anti- CD45.1 antibody | monoclonal | A20 | FITC | 1/200 | BD Biosciences | 553775 | FACS |
| CD45.1 | anti- CD45.1 antibody | monoclonal | A20 | PE-Cy7 | 1/200 | BioLegend | 110729 | FACS |
| CD45 | anti- CD45 antibody | monoclonal | 30-F11 | BV510 | 1/200 | BioLegend | 103138 | FACS |
| TCRβ | anti- TCR β chain antibody | monoclonal | H57-597 | PerCP-Cy5.5 | 1/200 | BD Biosciences | 109228 | FACS |
| TCRβ | anti- TCR β chain antibody | monoclonal | H57-597 | APC | 1/200 | BD Biosciences | 553174 | FACS |
| TCRβ | anti- TCR β chain antibody | monoclonal | H57-597 | APC-Cy7 | 1/200 | BioLegend | 109220 | FACS |
| B220 | anti- B220 antibody | monoclonal | RA3-6B2 | PerCP-Cy5.5 | 1/200 | BioLegend | 103236 | FACS |
| NK1.1 | anti- NK-1.1 antibody | monoclonal | PK136 | PE-Cy7 | 1/200 | BioLegend | 108714 | FACS |
| CD4 | anti- CD4 antibody | monoclonal | RM4-5 | FITC | 1/200 | BD Biosciences | 553047 | FACS |
| CD4 | anti- CD4 antibody | monoclonal | RM4-5 | BV510 | 1/200 | BD Biosciences | 563106 | FACS |
| CD8α | anti- CD8α antibody | monoclonal | 53-6.7 | FITC | 1/200 | BD Biosciences | 553031 | FACS |
| CD8α | anti- CD8α antibody | monoclonal | 53-6.7 | PerCP-Cy5.5 | 1/200 | BD Biosciences | 100734 | FACS |
| CD8α | anti- CD8α antibody | monoclonal | 53-6.7 | APC | 1/200 | BD Biosciences | 553035 | FACS |
| CD8α | anti- CD8α antibody | monoclonal | 53-6.7 | APC-Cy7 | 1/200 | BD Biosciences | 557654 | FACS |
| CD69 | anti- CD69 antibody | monoclonal | H1.2F3 | FITC | 1/200 | Thermo Fisher Scientific | 11-0691-85 | FACS |
| CD103 | anti- CD103 antibody | monoclonal | 2E7 | BV421 | 1/200 | BioLegend | 121421 | FACS |
| CXCR3 | anti- CXCR3 antibody | monoclonal | CXCR3-173 | APC | 1/200 | Thermo Fisher Scientific | 17-1831-82 | FACS |
| CXCR6 | anti- CXCR6 antibody | monoclonal | SA051D1 | APC | 1/200 | BioLegend | 151105 | FACS |
| CD62L | anti- CD62L antibody | monoclonal | MEL-14 | PE | 1/200 | BD Biosciences | 553151 | FACS |
| KLRG1 | anti- KLRG1 antibody | monoclonal | 2F1/KLRG1 | PerCP-Cy5.5 | 1/200 | BioLegend | 138417 | FACS |
| CD44 | anti- CD44 antibody | monoclonal | IM7 | BV421 | 1/200 | BioLegend | 103039 | FACS |
| CD11c | anti- CD11c antibody | monoclonal | HL3 | FITC | 1/200 | BD Biosciences | 557400 | FACS |
| CD11c | anti- CD11c antibody | monoclonal | HL3 | PE-Cy7 | 1/200 | BD Biosciences | 558079 | FACS |
| Foxp3 | anti- FOXP3 antibody | monoclonal | FJK-16s | PE | 1/200 | Thermo Fisher Scientific | 12-5773-82 | FACS |
| Foxp3 | anti- FOXP3 antibody | monoclonal | FJK-16s | PerCP-Cy5.5 | 1/200 | Thermo Fisher Scientific | 45-5773-82 | FACS |
| Helios | anti- Helios antibody | monoclonal | 22F-6 | APC | 1/200 | BioLegend | 137222 | FACS |
| TCRγδ | anti- TCR γδ chain antibody | monoclonal | GL3 | PerCP-Cy5.5 | 1/200 | BioLegend | 118118 | FACS |
| CD19 | anti- CD19 antibody | monoclonal | 1D3 | PE | 1/200 | BD Biosciences | 553786 | FACS |
| CD11b | anti- CD11b antibody | monoclonal | M1/70 | PE-Cy7 | 1/200 | BD Biosciences | 552850 | FACS |
| CD11b | anti- CD11b antibody | monoclonal | M1/70 | APC-Cy7 | 1/200 | BD Biosciences | 557657 | FACS |
| PDCA-1 | anti- PDCA-1 antibody | monoclonal | 129c1 | APC | 1/200 | BioLegend | 127106 | FACS |
| CCR5 | anti- CCR5 antibody | monoclonal | 7A4 | PE | 1/100 | Thermo Fisher Scientific | 12-1951-82 | FACS |
| Mouse BD Fc Block | anti- CD16/CD32 antibody | monoclonal | 2.4G2 | | 1/100 | BD Biosciences | 553141 | FACS |
| | Intracellular Fixation & Permeabilization Buffer Set | | | | | Thermo Fisher Scientific | 88-8824-00 | FACS |
| | Fixable Viability Dye eFluor 780 | | | | 1/1000 | Thermo Fisher Scientific | 65-0865-14 | FACS |
| | 7-AAD Viability Staining Solution | | | | 1/100 | BD Biosciences | 51-68981E | FACS |

Table S2 Koda et. al.

Supplementary table 3. List of Taqman probes used in this study

| Genes | catalog number | conjugate | vendor |
| --- | --- | --- | --- |
| *Col1a1* | Mm00801666_g1 | FAM | Themo Fisher Scientific |
| *Col1a2* | Mm00483888_m1 | FAM | Themo Fisher Scientific |
| *Acta2* | Mm00725412_s1 | FAM | Themo Fisher Scientific |
| *Timp1* | Mm01341361_m1 | FAM | Themo Fisher Scientific |
| *Desmin* | Mm00802455_m1 | FAM | Themo Fisher Scientific |
| *Spp1* | Mm00436767_m1 | FAM | Themo Fisher Scientific |
| *Il15* | Mm00434210_m1 | FAM | Themo Fisher Scientific |
| *Ccl3* | Mm00441258_m1 | FAM | Themo Fisher Scientific |
| *Ccl4* | Mm00443111_m1 | FAM | Themo Fisher Scientific |
| *Ccl5* | Mm01302427_m1 | FAM | Themo Fisher Scientific |
| *Ccr5* | Mm001216171_m1 | FAM | Themo Fisher Scientific |
| *Fas* | Mm00433237_m1 | FAM | Themo Fisher Scientific |
| *Gapdh* | Mm99999915_g1 | VIC | Themo Fisher Scientific |

Table S3 Koda et. al.
