# Supplementary material for: CD8+ tissue-resident memory T cells promote liver fibrosis resolution by inducing apoptosis of hepatic stellate cells
Source: Nat Commun. 2021 Jul 22;12:4474. doi: 10.1038/s41467-021-24734-0 (PMC8298513; doi:10.1038/s41467-021-24734-0)
Supplement: Supplementary file 2 — Reporting Summary [file 41467_2021_24734_MOESM2_ESM.pdf]

nature research

Corresponding author(s): Nobuhiro Nakamoto, Takanori Kanai

Last updated by author(s): Jun 14, 2021

Reporting Summary

Nature Research wishes to improve the reproducibility of the work that we publish. This form provides structure for consistency and transparency in reporting. For further information on Nature Research policies, see our Editorial Policies and the Editorial Policy Checklist.

Statistics

For all statistical analyses, confirm that the following items are present in the figure legend, table legend, main text, or Methods section.

n/a	Confirmed	
☐	☒	The exact sample size (n) for each experimental group/condition, given as a discrete number and unit of measurement
☐	☒	A statement on whether measurements were taken from distinct samples or whether the same sample was measured repeatedly
☐	☒	The statistical test(s) used AND whether they are one- or two-sided<br>*Only common tests should be described solely by name; describe more complex techniques in the Methods section.*
☐	☒	A description of all covariates tested
☐	☒	A description of any assumptions or corrections, such as tests of normality and adjustment for multiple comparisons
☐	☒	A full description of the statistical parameters including central tendency (e.g. means) or other basic estimates (e.g. regression coefficient) AND variation (e.g. standard deviation) or associated estimates of uncertainty (e.g. confidence intervals)
☐	☒	For null hypothesis testing, the test statistic (e.g. F, t, r) with confidence intervals, effect sizes, degrees of freedom and P value noted<br>*Give P values as exact values whenever suitable.*
☒	☐	For Bayesian analysis, information on the choice of priors and Markov chain Monte Carlo settings
☒	☐	For hierarchical and complex designs, identification of the appropriate level for tests and full reporting of outcomes
☒	☐	Estimates of effect sizes (e.g. Cohen's d, Pearson's r), indicating how they were calculated

Our web collection on statistics for biologists contains articles on many of the points above.

Software and code

Policy information about availability of computer code

Data collection	BD FACSDiva version 8.0.1 for flowcytometry.
Data analysis	FlowJo v10 (Flow Jo LLC) for flowcytometry, Prism v7 and v8 (Graphpad) for the statistics, salmon v0.14.1 and seurat v3.1.1 for RNA-seq. Our script to analyse and visualize the scRNA/TCR-seq in this study has faithfully followed Satija's vignettes (https://satijalab.org/seurat/articles/integration_introduction.html). For vdj analysis, we used a custom code, which should have been linked to the original resource (https://ucdavis-bioinformatics-training.github.io/2020-Advanced_Single_Cell_RNA_Seq/data_analysis/VDJ_Analysis_fixed).

For manuscripts utilizing custom algorithms or software that are central to the research but not yet described in published literature, software must be made available to editors and reviewers. We strongly encourage code deposition in a community repository (e.g. GitHub). See the Nature Research guidelines for submitting code & software for further information.

Data

Policy information about availability of data

All manuscripts must include a data availability statement. This statement should provide the following information, where applicable:

- Accession codes, unique identifiers, or web links for publicly available datasets
- A list of figures that have associated raw data
- A description of any restrictions on data availability

The raw scRNA sequencing data have been deposited in the NCBI GEO database under accession number GSE176210 (https://www.ncbi.nlm.nih.gov/geo/query/acc.cgi?acc=GSE176210). The raw bulk-RNA sequencing data have been deposited in the DNA Data Bank of Japan under accession number DRA012190 (https://www.ncbi.nlm.nih.gov/sra/?term=DRA012190). Source data are provided with this paper as a Source Data file. All other data are available from the corresponding authors upon reasonable request.

Field-specific reporting

Please select the one below that is the best fit for your research. If you are not sure, read the appropriate sections before making your selection.

☒ Life sciences ☐ Behavioural & social sciences ☐ Ecological, evolutionary & environmental sciences

For a reference copy of the document with all sections, see nature.com/documents/nr-reporting-summary-flat.pdf

Life sciences study design

All studies must disclose on these points even when the disclosure is negative.

Sample size	No statistical methods were used to predetermine sample sizes. Sample sizes were determined based on previous studies from our group (J Clin Invest. 2018 Apr 2;128(4):1581-1596 and J Hepatol. 2021 Mar;74(3):511-521.) and publications in the field.
Data exclusions	No data were excluded from the analysis.
Replication	Each experiment was replicated more than two times with reproducible results, as indicated in a 'Statistics and reproducibility' section.
Randomization	Mice were matched based on gender (male), age, weight, and serum ALT levels, and randomly allocated to each group. For human study, the participants were randomly allocated to the group based on the histological findings.
Blinding	The data collection and analysis were not blinded. Blinding was not possible as the investigators were also conducting the experiments and had to be aware of controls and treated groups.

Reporting for specific materials, systems and methods

We require information from authors about some types of materials, experimental systems and methods used in many studies. Here, indicate whether each material, system or method listed is relevant to your study. If you are not sure if a list item applies to your research, read the appropriate section before selecting a response.

Materials & experimental systems

n/a	Involved in the study	
☐	☒	Antibodies
☒	☐	Eukaryotic cell lines
☒	☐	Palaeontology and archaeology
☐	☒	Animals and other organisms
☐	☒	Human research participants
☒	☐	Clinical data
☒	☐	Dual use research of concern

Methods

n/a	Involved in the study	
☒	☐	ChIP-seq
☐	☒	Flow cytometry
☒	☐	MRI-based neuroimaging

Antibodies

Antibodies used	For flow cytometry analysis, the following antibodies were used in this study. anti-mouse CD45 (BioLegend, BV510, clone 30-F11, cat 103138, 1/200), anti-mouse CD45.2 (BD BioLegend FITC/BV510, clone 104, cat 553772/109838, 1/200), anti-mouse CD45.1 (BD Biosciences/BioLegend, FITC/PE-Cy7, clone A20, cat 553775/110729, 1/200), anti-mouse TCR-β (BD Biosciences/BioLegend, PerCP-Cy5.5/APC/APC-Cy7, clone H57-597, cat 109228/553174/109220, 1/200), anti-mouse NK1.1 (BioLegend, PE-Cy7, clone PK136, cat 108714, 1/200), anti-mouse CD4 (BD Biosciences, FITC/BV510, clone RM4-5, cat 553047/563106, 1/200), anti-mouse CD8α (BD Biosciences, FITC/PerCP-Cy5.5/APC/APC-Cy7, clone 53-6.7, cat 100734/553035/557654, 1/200), anti-mouse CD69 (Thermo Fisher Scientific, FITC, clone H1.2F3, cat 11-0691-85, 1/200), anti-mouse CD103 (Biolegend, BV421, clone 2E7, cat 121421, 1/200), anti-mouse CXCR3 antibody (Thermo Fisher Scientific, APC, clone CXCR3-173, cat 17-1831-82, 1/200), anti-mouse CXCR6 (BioLegend, APC, clone SA051D1, cat 151105, 1/200), anti-mouse CD62L (BD Biosciences, PE, clone MEL-14, cat 553151, 1/200), anti-mouse KLRG1 (BioLegend, PerCP-Cy5.5, clone 2F1/KLRG1, cat 138417, 1/200), anti-mouse CD44 (BioLegend, BV421, clone IM7, cat 103039, 1/200), anti-mouse Foxp3 (Thermo Fisher Scientific, PE/PerCP-Cy5.5, clone FJK-16s, cat 12-5773-82, 45-5773-82, 1/100), anti-mouse Helios (BioLegend, APC, clone 22F6, cat 137222, 1/100), anti-mouse TCRγδ (BD Biosciences, PerCP-Cy5.5, clone GL3, cat 118118, 1/200), anti-mouse CD19 (BD Biosciences, PE, clone 1D3, 553876, 1/200), anti-mouse CD11b (BD Biosciences, PE-Cy7/APC-Cy7, clone M1/70, 552850/557657, 1/200), anti-mouse CD11c (BD Biosciences, FITC/PE-Cy7, clone HL3, cat 557400/558079, 1/200), anti-mouse B220 (BioLegend, PerCP-Cy5.5, clone RA3-6B2, cat 103236, 1/200), anti-mouse PDCA-1 (BioLegend, APC, clone 129c1, cat 127106, 1/200), anti-mouse CCR5 (Thermo Fisher Scientific, PE, clone 7A4, cat 12-1951-82, 1/100). For immunohistochemistry, the following antibodies were used in this study. anti-mouse CD8 antibody (Santa Cruz, clone 6A242, cat sc-70802, 1/50), anti-mouse desmin antibody (abcam, clone DE-U-10, cat ab15200, 1/100), anti-mouse desmin antibody (abcam, cat ab6322, 1/100), anti-mouse cleaved caspase 3 antibody (R & D systems, clone 269518, cat MAB835, 1/100) , anti- mouse FasL antibody (sinobiological, cat 101984, 1/100) , anti-mouse Fas antibody (R & D systems, cat AF435, 1/100), anti-mouse Granzyme B antibody (R & D systems, AF1865, 1/100), and anti-mouse Perforin antibody (Abcam, clone CB5.4, cat Ab16074, 1/100), Anti-human CD8 monoclonal Ab (Nichirei, clone C8/144B, cat 413211, 1/100), and anti-human CD69 monoclonal antibody (Abcam, clone EPR21814, cat ab233396, 1/100). For in vivo

	assay, the following antibodies were used in this study. anti-mouse CD8α antibody (Bioxcell, clone 2.43, cat BE0061), anti-mouse NK1.1 antibody (Bioxcell, clone PK136, cat BE0036), anti-mouse CD4 antibody (Bioxcell, clone GK1.5, cat BP0003-1), anti-mouse IL-15 antibody (Bioxcell, clone AIO.3, cat BE0315), anti-mouse FasL antibody (Bioxcell, clone MFL3, cat BE0319), and anti-mouse CXCR3 antibody (Bioxcell, clone CXCR3-173, cat BE0249). For in vivo assay, the following antibodies were used in this study. anti-mouse FasL (1 μg/mL; BioLegend, clone MFL4, cat 106707), anti-mouse IFN-γ Ab (1 μg/mL; BD Biosciences Pharmingen, clone XMG1.2, cat 559065), anti-mouse PD-1 Ab (1 μg/mL; BioLegend, clone 29F.1A12, cat 135246), anti-mouse TIGIT Ab (1 μg/mL; BioLegend, clone A17200C, cat 622203).<br>This information is summarised in Supplementary Table 2.
Validation	All the antibodies used in this study were commercial antibodies and were only used for applications, with validation procedures described on the following sites of the manufacturers:<br>https://www.thermofisher.com; https://www.biolegend.com; https://www.abcam.com; https://www.bdbiosciences.com; https://www.rndsystems.com; https://www.sinobiological.com; https://www.nichirei.co.jp/bio.

Animals and other organisms

Policy information about studies involving animals; ARRIVE guidelines recommended for reporting animal research

Laboratory animals	C57BL/6J mice (Ly5.2) were purchased from CLEA Japan, INC. (Tokyo, Japan). Ly5.1 mice were obtained from The Jackson Laboratory (Maine, USA). B6.Itgax-dtr/dtr mice were provided by Dr. Kenya Honda (Keio University). B6.Siglech-dtr/dtr mice were provided by Dr. Katsuaki Sato (Miyazaki University). All mice were maintained under specific pathogen–free (SPF) conditions with a 12h light/dark cycle, at a temperature of 22-25 °C and a relative humidity of 45-55 % in the Animal Care Facility of Keio University School of Medicine. Male mice, aged 6 to 12 weeks and weighing 18 to 25 g, were used in all experiments.
Wild animals	This study did not involve wild animals.
Field-collected samples	This study did not involve samples collected from the field.
Ethics oversight	Animal Ethics Committee of Keio University approved all animal studies.

Note that full information on the approval of the study protocol must also be provided in the manuscript.

Human research participants

Policy information about studies involving human research participants

Population characteristics	Liver tissue samples were obtained from patients with NASH (n = 13) and patients suffering from hepatic metastasis of gastrointestinal cancer with normal liver function (n = 5) as the control group.<br>Clinical characteristics and baseline demographics are presented in Supplementary Table S1.
Recruitment	The participants were recruited with written informed consent. There is no indication that self selection bias affected any of the results.
Ethics oversight	The institutional review board of Keio University School of Medicine approved all human studies (No. 20120395 and No. 20040034) according to the guidelines of the 1975 Declaration of Helsinki (2008 revision).

Note that full information on the approval of the study protocol must also be provided in the manuscript.

Flow Cytometry

Plots

Confirm that:

- [x] The axis labels state the marker and fluorochrome used (e.g. CD4-FITC).
- [x] The axis scales are clearly visible. Include numbers along axes only for bottom left plot of group (a 'group' is an analysis of identical markers).
- [x] All plots are contour plots with outliers or pseudocolor plots.
- [x] A numerical value for number of cells or percentage (with statistics) is provided.

Methodology

Sample preparation	livers were perfused through the postcaval vein with HBSS (Nacalai Tesque, Kyoto, Japan) and then minced and homogenized by gentle MACS (Miltenyii biotec japan, Tokyo, Japan). The suspensions were passed through 100 μm nylon mesh, centrifuged at 50 g for 5 minute, and the supernatant was washed once. Cells were suspended in 40% Percoll and overlaid on a 75% Percoll fraction. Percoll gradient separation was performed by centrifugation at 840 g for 20 min at room temperature. MNCs were collected at Aldrich , MO, USA) supplemented with 10% fetal bovine serum and 1% penicillin/streptomycin (Nacalai Tesque). The numbers of live cells were determined by Countess II (Thermo Fisher Scientific).
Instrument	Isolated cells were stained with fluorochrome-conjugated antibody.<br>For cell sorting: FACSAria II (Becton Dickinson).<br>For analysis: FACSCanto II or FACSFortessa X-20 (Becton Dickinson).

Software	FACSDiva for collection and FlowJo (v10) for analysis
Cell population abundance	Populations were validated for purity by a post-sort analysis by FACS.
Gating strategy	Gating strategies are reported in Supplementary Figure1h and 1i.

☒ Tick this box to confirm that a figure exemplifying the gating strategy is provided in the Supplementary Information.
